# Single-cell quantification of a broad RNA spectrum reveals unique noncoding patterns associated with cell types and states

**DOI:** 10.1073/pnas.2113568118

**Published:** 2021-12-15

**Authors:** Alina Isakova, Norma Neff, Stephen R. Quake

**Affiliations:** ^a^Department of Bioengineering, Stanford University, Stanford, CA 94305;; ^b^Chan Zuckerberg Biohub, San Francisco, CA 94158;; ^c^Department of Applied Physics, Stanford University, Stanford, CA 94305

**Keywords:** single-cell RNA-seq, noncoding RNA, cell cycle, differentiation

## Abstract

The ability to interrogate total RNA content of single cells would enable better mapping of the transcriptional logic behind emerging cell types and states. However, current single-cell RNA-sequencing (RNA-seq) methods are unable to simultaneously monitor all forms of RNA transcripts at the single-cell level, and thus deliver only a partial snapshot of the cellular RNAome. Here we describe Smart-seq-total, a method capable of assaying a broad spectrum of coding and noncoding RNA from a single cell. Smart-seq-total does not require splitting the RNA content of a cell and allows the incorporation of unique molecular identifiers into short and long RNA molecules for absolute quantification. It outperforms current poly(A)-independent total RNA-seq protocols by capturing transcripts of a broad size range, thus enabling simultaneous analysis of protein-coding, long-noncoding, microRNA, and other noncoding RNA transcripts from single cells. We used Smart-seq-total to analyze the total RNAome of human primary fibroblasts, HEK293T, and MCF7 cells, as well as that of induced murine embryonic stem cells differentiated into embryoid bodies. By analyzing the coexpression patterns of both noncoding RNA and mRNA from the same cell, we were able to discover new roles of noncoding RNA throughout essential processes, such as cell cycle and lineage commitment during embryonic development. Moreover, we show that independent classes of short-noncoding RNA can be used to determine cell-type identity.

## Significance

Each individual cell transcribes nearly 85% of the genome. However, when it comes to the analysis of cellular RNA, most studies are still only looking at the 3% that correspond to protein-coding transcripts. The role and abundance of the remaining RNAs across individual cells remain largely unknown. In the present study we describe Smart-seq-total, an RNA-sequencing method that delivers a broad picture of the total cellular RNA content. Using Smart-seq-total, we analyzed the content of hundreds of human and mouse cells and showed that the noncoding RNA content of cells significantly differs across cell types and dynamically changes throughout the vital processes of a cell, such as cell cycle and cell differentiation.

Efforts in characterizing transcriptional states of single cells have so far mostly focused on protein-coding RNA (1–4). However, a growing number of studies indicate that noncoding RNAs (ncRNAs), are actively involved in cell function and specialization (5–8). Importantly, compared to the coding RNA, which is transcribed from only ∼1 to 2% of the genome, ncRNA constitutes a major fraction of all cellular transcripts and covers ∼70% of the genomic content (9). The role of these transcripts in shaping different cell types and states remain poorly understood.

Several groups have developed techniques to measure ncRNA in single cells (10–15). The respective methods, however, are designed to target only a subset of noncoding transcripts, which are either short (∼18 to 200 nt; e.g., microRNA) (11, 16), long (>200 nt, e.g., long ncRNA [lncRNA] or circular RNA [circRNA]) (10, 14, 17, 18), or limited to specific types of RNA molecules, such as miRNA–mRNA pairs, for example (10, 12). None of the existing methods are able to simultaneously quantify all RNA types within a cell. This limits the ability to map the regulatory connection between coding and noncoding transcripts within a cell and motivates the need for the development of novel single-cell technologies capable of assaying both poly(A)^+^ and poly(A)^−^ RNA, irrespective of transcript length.

In the present study we describe Smart-seq-total, a scalable one-pot method designed to capture both coding and noncoding transcripts regardless of their length. Inspired by the widely used Smart-seq2 protocol (19), this method harnesses the template-switching capability of MMLV reverse transcriptase to generate full-length cDNA with high yield and quality. Smart-seq-total captures nonpolyadenylated RNA through template-independent addition of polyA tails and further oligo-dT priming of all cellular transcripts. Because all RNA molecules are reverse transcribed using oligo-dT, they can also be tagged with unique molecular identifiers (UMIs) at that step. Therefore, Smart-seq-total simultaneously quantifies the levels of mRNA alongside other RNA types in the same cell, which permits: 1) the annotation of cell types and states based on mRNA and integration of this data with the existent single-cell RNA-sequencing (scRNA-seq) datasets, and 2) the discovery of noncoding regulatory patterns of the respective states.

## Results

**Fig. 1.**
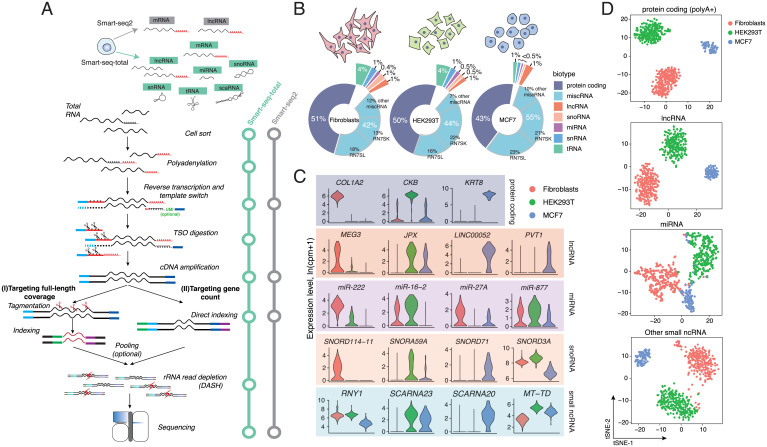
Smart-seq-total performance. (*A*) Schematic comparison of Smart-seq2 and Smart-seq-total pipelines. Following cell lysis, total cellular RNA is polyadenylated, primed with anchored oligodT, and reverse transcribed in a presence of the custom degradable TSO. After reverse transcription, TSO is enzymatically cleaved, single-stranded cDNA is amplified and cleaned up. Amplified cDNA is then either tagmented or directly indexed, pooled, and depleted from ribosomal sequences using DASH (23). The resulting indexed libraries are then pooled and sequenced on Illumina platform. (*B*) Distribution of mapped reads across RNA types in human primary fibroblasts, HEK293T, and MCF7 cells. Percentage of total reads mapped to each RNA type. miscRNA class is additionally split into RN7SK, RN7SL, and other miscRNA categories. (*C*) Examples of coding and noncoding marker genes for each cell type. Top exemplary markers per biotype computed among cell types using Wilcoxon rank sum test. *RNY1* belongs to miscRNA, *SCARNA23* and *SCARNA20* to scaRNA, *MT-TD* to mitochondrial tRNA class. (*D*) t-SNE plots of three profiled human cell types generated using indicated subset of genes. From top to bottom: protein coding, lncRNA, miRNA, and other small ncRNA (include snoRNA, snRNA, scaRNA, scRNA, and miscRNA). We have excluded histone coding genes from the protein coding (polyA+) set, since a large fraction of these RNAs are known to lack polyA tails (60).

Smart-seq-total relies on the ability of *Escherichia coli* poly(A) polymerase to add adenine tails to the 3′ prime of RNA molecules. Total polyadenylated RNA is then reverse-transcribed using anchored oligo dT, in the presence of the template switch oligo (TSO) (20) (Fig. 1*A*). Compared to previous studies that explored similar approaches to construct libraries from total RNAs (21, 22), Smart-seq-total utilizes an optimized version of the TSO (19), specifically engineered to be rapidly eliminated from the reaction through enzymatic digestion directly following the reverse transcription. This allows us to remove the “contaminant” constructs, originating from polyA-tailing and mispriming of TSO, which otherwise dominate the resulting sequencing library and render the short RNA transcripts undetectable (*SI Appendix*, Fig. S1 *A* and *B*). Additionally, we employ a CRISPR-mediated removal of overrepresented sequences, which allows us to eliminate the majority of the sequences corresponding to ribosomal RNA from the final library in a single-pool reaction (23) (targeting rRNA regions and other abundant RNA molecules) (*SI Appendix*, Fig. S1 *A* and *B* and Table S1).

When applied to single HEK293T cells, Smart-seq-total identified the full complement of mRNA as well as a broad spectrum of ncRNA genes, such as small nucleolar RNA (snoRNA), small Cajal body-specific RNA (scaRNA), histone RNA, and lncRNA. The majority of these molecules endogenously lack poly(A) tails and thus cannot be captured through a direct polyA-priming employed by Smart-seq2 (1) or other popular scRNA-seq methods (*SI Appendix*, Fig. S1 *C* and *D*). Among other ncRNA detected by Smart-seq-total are transfer RNAs (tRNAs) and mature miRNAs (*SI Appendix*, Fig. S1 *C* and *D*). To facilitate the quantitative comparison across RNA biotypes within a cell, we also implemented a UMI-compatible version of Smart-seq-total (v2) (*SI Appendix*, Fig. S2 *A*–*C*). Like Smart-seq3, Smart-seq-total (v2) does not require a clean-up step after reverse transcription and thus is also potentially subject to random UMI incorporation during cDNA amplification (24). We show, however, that under the current protocol conditions these events are negligible (*SI Appendix*, Fig. S2 *D*–*F*). Both versions of Smart-seq-total are compatible with the Tn5-based fragmentation of cDNA in order to sequence full-length transcript coverage (*SI Appendix*, Fig. S3). The sensitivity of Smart-seq-total estimated based on external RNA control consortium (ERCC) capture is comparable to Smart-seq2 (*SI Appendix*, Fig. S3 *E* and *F*) (25). At the same time, Smart-seq-total detects a broader spectrum of RNA types than previous single-cell approaches and furthermore allows the incorporation of UMIs for absolute quantitation into both short and long RNA molecules (*SI Appendix*, Figs. S1 and S4).

To demonstrate the scalability of the method, we sequenced total RNA from individual human primary dermal fibroblasts (*n* = 277), HEK293T (*n* = 245), and MCF7 (*n* = 90) cells sorted in 384-well plates and processed in one-tenth of the standard Smart-seq2 volume (i.e., cells are sorted in 0.3 μL of lysis buffer and RNA is reverse-transcribed in 1 μL) (*Materials and Methods*) (19). We sequenced the libraries to obtain one to two million reads per cell, mapped, and counted the reads using the reference that contains both coding and noncoding transcripts (including miRNA, snoRNA, small nuclear RNA [snRNA], tRNA, and so forth). Within all three cell types, we identified a broad spectrum of transcripts, such as mRNA, miRNA, lncRNA, and snoRNA in each profiled cell (Fig. 1*B* and *SI Appendix*, Fig. S5). We found metazoan RNA7SK and RN7SL1, which are involved in regulation of transcription and translation, respectively (annotated as “miscellaneous RNA”-type [miscRNA] in the GENCODE database) to be the most abundant in our data comprising together ∼40% of all mapped reads (Fig. 1*B* and *SI Appendix*, Fig. S6). We demonstrate that, if desired, these molecules could also be depleted from the sequencing libraries at the rRNA depletion step using dedicated CRISPR guides (*SI Appendix*, Fig. S1 *A* and *B* and Table S1). Among cell-type–specific transcripts, we found well-characterized marker genes for either fibroblasts (*COL1A2*, *FN1, MEG3*), HEK293T (*CKB, AMOT, HEY1*), or MCF7 cells (*KRT8, TFF1*) (Fig. 1*C* and *SI Appendix*, Fig. S8*A*), as well as transcripts that belong to various types of ncRNA, such as microRNA, snoRNA, and lncRNA (Fig. 1*C* and *SI Appendix*, Fig. S8). For example, we found high levels of *MIR222* in fibroblasts while we could not detect it in MCF7 cells. We also observed that oncogenic miRNA cluster *MIR17HG* is specific to HEK293T cells, while not found in either fibroblasts or MCF7 cells. In contrast, MCF7-specific transcripts include lncRNA, such as *LINC00052*, as well as snoRNA, such as *SNORD71* and *SNORD104*.

Given the observed differences in the levels of ncRNA across profiled cells, we next asked whether ncRNA alone could be used to distinguish cell types. To answer this question, we performed principal component analysis (PCA) followed by the dimensionality reduction through t-distributed stochastic neighbor embedding (t-SNE) on the genes corresponding to one or multiple ncRNA types. Evaluation of the similarity between cells in two-dimensional space revealed that, in addition to lncRNA (26), miRNA taken alone separate the investigated cell types into three distinct clusters. Combining snoRNA, scaRNA, snRNA, and tRNA together allowed us to achieve similar results (Fig. 1*D*). While the exact function of individual snoRNA and scaRNA remains largely uncharacterized, these RNAs are believed to play a vital role in posttranscriptional and posttranslation control (27–29). Here we show that their abundance is also cell-type specific (*SI Appendix*, Fig. S8*A*).

**Fig. 2.**
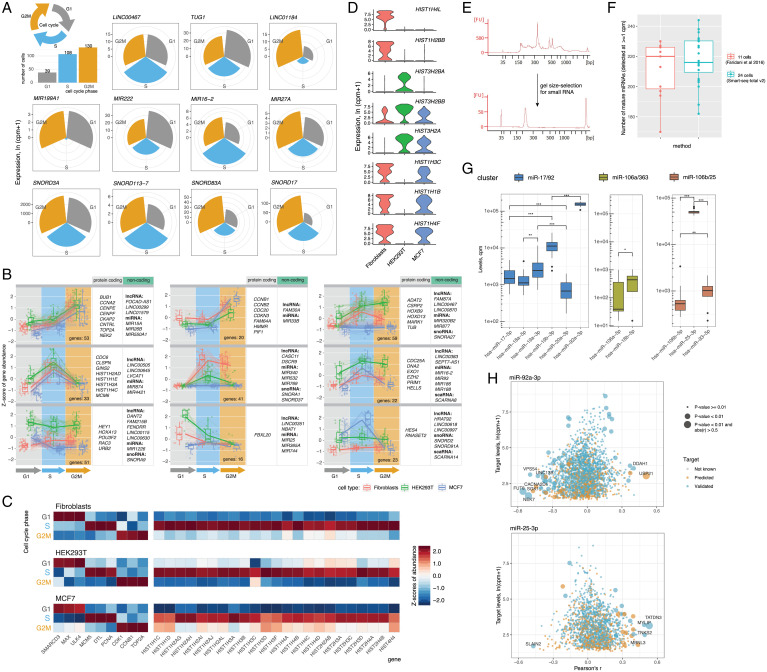
Dynamics of cellular noncoding transcripts throughout the cell cycle. (*A*) Cell-cycle–dependent expression of noncoding genes. Examples of lncRNA, miRNA, and snoRNA differentially expressed throughout the cell cycle in human primary dermal fibroblasts. Circular charts depict average expression of a given gene across all cells identified to be in a certain phase of the cell cycle. (*B*) Cell-cycle–specific gene clusters comprised of coding and noncoding RNA. Clusters were identified through hierarchical clustering of top 750 mRNA differentially expressed during the cell cycle and all noncoding genes expressed in at least one phase. (*C*) Expression of known cell-cycle and histone genes across G1, S, and G2M phases. A curated list of histone RNA detected in all three cell types is shown. (*D*) Examples of histone mRNA differentially expressed between three profiled cell types. Top three marker histone genes per cell type are shown. (*E*) Size-selection of small RNA library fraction. Bioanalyzer traces corresponding to UMI-tagged and indexed Smart-seq-total v2 library (*Upper*) and a size-selected library containing small RNA (*Lower*). (*F*) Number of detected miRNA. Comparison of miRNA detection by Smart-seq-total v2 and a dedicated single-cell small RNA-seq method (11). (*G*) Cellular levels of mature miRNA members of miR-17/92, miR-106a/363 and miR106b/25 clusters. Asterisks indicate the significance level estimated from Wicoxon rank sum test: ***<= 0.001,**<= 0.01,*<= 0.05 *P* value respectively. (*H*) Pair-wise correlation scores computed for miR-92a-3p and miR-25-3p and their respective mRNA targets (both predicted and validated) across ∼300 HEK293T cells. *P* values of correlations were computed using *t* test and adjusted using Benjamini–Hochberg correction).

After binning all the cells according to cell-cycle phases (30), we observed that in addition to cell-type–dependent differences in ncRNA, the abundance of certain noncoding transcripts also changed throughout the cell cycle (Fig. 2*A*). In agreement with previous bulk studies, which suggested the involvement or miRNA in cell-cycle regulation (31, 32), we found that levels of a subset of miRNAs in a cell dynamically change through the cell cycle, peaking at either S, G2M, or G1 phase (Fig. 2*A*). For example, our data showed that the levels of *MIR16-2* in fibroblasts are high during the S phase and later gradually decrease during G2M and G1 phases (*SI Appendix*, Fig. S9). The opposite holds true for *MIR222*, in both fibroblasts and HEK293T cells, which is more abundant during cell proliferation (G1) and decays during DNA replication (S) and cell division (G2M) phases (Fig. 2*A* and *SI Appendix*, Fig. S10). Among miRNAs more abundant during G2M phase, we identified *MIR27A*, *MIR103A2*, and *MIR877* (*SI Appendix*, Figs. S9–S11). In addition to miRNA, a large number of lncRNA, snRNA, scaRNA, snoRNA, and miscRNA were also up-regulated (log_2_ fold-change [FC] > 1, adjusted *P* < 0.01) during the G2M phase (Fig. 2*A* and *SI Appendix*, Figs. S9–S11). Given the active role of these RNA types in splicing and ribosome biogenesis, we suggest that they are produced by the cell in response to a rapid demand for protein synthesis and cell growth during the G2M phase.

To further link the observed ncRNA dynamics with the expression of well-characterized cell-cycle mRNA markers, we searched for coregulated coding and noncoding genes throughout the cell cycle. We identified 24 clusters comprised of coexpressed coding and noncoding genes specific to either one or multiple cell types (Fig. 2*B*, *SI Appendix*, Fig. S12, and Dataset S1). Two of these mixed-gene clusters (33 genes up-regulated in the S phase and 53 genes up-regulated in the G2M phase) showed identical patterns in all three profiled cell types. Interestingly, both clusters are marked by landmark cell-cycle genes—such as *CCNA2*, *MCM6*, and *TOP2A*—but also include miRNAs, lncRNAs, and snRNAs previously unknown to follow a distinct expression pattern upon transition between phases.

Histone RNA is another type of mainly nonpolyadenylated RNA, which we observed to be strongly correlated with the cell cycle. Consistent with prior studies (33, 34), histone RNA levels sharply rise during the S phase in all three profiled cell types (Fig. 2*C*). The ability to capture nonpolyadenylated histones also has a strong impact on cell clustering, by introducing a cell-cycle bias. Particularly, histones drive the separation of each cell type into two distinct populations (*SI Appendix*, Fig. S13*A*), marked by increased levels of the majority of histone genes during the DNA replication phase (*SI Appendix*, Fig. S13*B*).

In addition to being expressed in a cell-cycle–dependent manner, we also identified several histones to be cell-type specific. For example, *HIST1H4L* is expressed in fibroblasts but absent in HEK293T and MCF7 cells, while *HIST1H1B* is absent in HEK293T cells while present in the other two cell types (Fig. 2*D*). Given the importance of histones in establishing and maintaining a distinct chromatin landscape of a cell, we anticipate that the ability to measure corresponding transcripts could be valuable for predicting the epigenetic state of a cell.

In principle, Smart-seq-total is designed to broadly quantify total cellular RNA content. However, we also show that short, less abundant molecules, such as miRNAs (35), can be size-selected from a UMI-tagged and indexed Smart-seq-total library for further in-depth analysis (Fig. 2*E*). In the example of 24 HEK293T cells we demonstrate that this size-based enrichment strategy yields results comparable to the state-of-the-art single-cell small RNA-seq method (11) in terms of the number and type of miRNAs detected per cell (Fig. 2*F* and *SI Appendix*, Fig. S14 *A*–*C*). Among the most abundant miRNAs in profiled HEK293T cells, we identified multiple members of the three conserved paralog clusters—miR-17/92, miR106a/363, and miR-10b/25 (36)—as well as various members of the let-7 miRNA family (*SI Appendix*). We noted that levels of mature miRNAs generally correlated better within a cluster rather than across different clusters (*SI Appendix*, Fig. S14 *D*–*F*). However, the abundance of mature forms largely varied across cluster members. Specifically, we found that the levels of two mature miRNAs, miR-92a-3p and miR-25-3p, were several folds higher than those of any other cluster member (Fig. 2*G*). Selective retention of miR-92a was previously observed at the tissue level in vivo (36) and has been attributed to differential posttranscriptional processing of cluster members (37, 38). Our data indicate that the phenomenon of selective miRNA retention can be observed at the single-cell level and that it extends beyond the 17/92 cluster.

While short RNAs are selected from a fully constructed UMI-indexed Smart-seq-total library, the remaining total library can be used to quantify mRNA counterpart of the same cells. We applied this strategy to investigate the relationship of the retained miR-92a-3p and miR-25-3p with the rest of the transcriptome at the physiological cell state. Specifically, we generated a matching miRNA–mRNA profile for ∼300 HEK 293T cells by sequencing the complete Smart-seq-total library, as well as its small RNA fraction (*SI Appendix*). We used the obtained data to perform a pair-wise correlation of miR-92a-3p and miR-25-3p with the rest of the genes across profiled cells. Among the most correlated and anticorrelated genes (Pearson’s *r* <−0.5 or > 0.5, adjusted *P* < 0.01), we identified several validated targets of the respective miRNAs (Fig. 2*H* and *SI Appendix*, Fig. S15*A*). Interestingly, we also found that several other genes, which are neither known nor predicted to directly interact with the respective miRNAs, correlate with either miR-92a-3p, miR-25-3p or both ([abs(r)] > 0.5, adjusted *P* value <0.01) (*SI Appendix*, Fig. S15*A* and Dataset S2). Gene ontology (GO) analysis on top correlated and anticorrelated genes (*Materials and Methods*) revealed energy metabolism as well as RNA translation among their main molecular functions (*SI Appendix*, Fig. S15*B*). The MiR-17/92 cluster has been previously shown to regulate tumor metabolic reprogramming (39). The results of our miRNA–mRNA coanalysis align with those findings and indicate that miR-92a-3p and miR-25-3p miRNA levels are linked to the expression of a metabolic gene set at the physiological cell state.

**Fig. 3.**
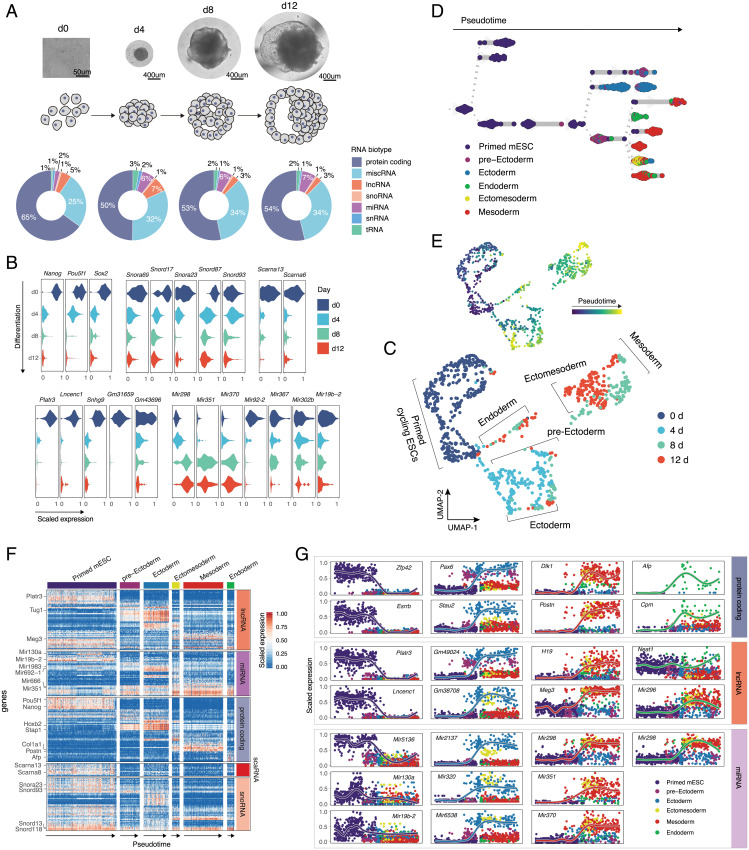
Coding and noncoding signature of differentiated single mESCs. (*A*) Microscope images and corresponding schematic representations of EB formation at four sampled time points. Pie charts represent distribution of mapped reads across RNA types. Genes were assigned to a specific biotype based on GENCODE M23 annotation for the reference chromosomes. tRNA was quantified by mapping the reads, nonmapping to any other RNA type, to the high-confidence gene set obtained from GtRNAdb. (*B*) Exemplary coding and noncoding genes that are up- or down-regulated during EB formation. Subpanels are grouped according to RNA type. (*C*) UMAP plot of collected cells colored by timepoint. Cells were clustered using a *k* nearest-neighbor algorithm and cell lineages were annotated based on the expression of marker genes within the identified clusters. (*D*) Lineage tree of EB differentiation. Each dot represents a cell colored according to the assigned lineage. Cells are arranged according to the computed pseudotime. (*E*) UMAP plot of collected cells colored by pseudotime. (*F*) Heatmap showing the variability in coding and noncoding gene expression across identified clusters. (*G*) Temporal and lineage-specific expression of selected protein-coding, lncRNA, and miRNA genes. Each column from left to right shows genes specific to: pluripotency state, ectoderm, mesoderm, or endoderm lineages.

Finally, we sought to understand whether the unique noncoding signature acquired by different cell types is established during early stages of cell development, and if so, how dynamic it is with respect to cellular transcriptome. To address this question, we referred to an in vitro model of early lineage commitment: the differentiation of pluripotent stem cells into embryoid bodies (EBs) (40). The role of ncRNA in maintaining stem cell pluripotency and lineage commitment has been demonstrated previously through bulk experiments (41, 42). Thus, we hypothesized that applying Smart-seq-total to single cells at different stages of EB formation would allow us to identify coexpressed coding and noncoding transcripts within emerging lineages. As such, we analyzed the RNAome of primed pluripotent stem cells and that of individual cells obtained from dissociated EB at days 4, 8, and 12 of culture (∼200 cells per each time point, 913 cells in total) (Fig. 3*A*). Consistent with previous studies (43), the number of coding genes expressed by pluripotent stem cells was also higher compared to differentiated progenitors (*SI Appendix*, Fig. S16*A*). This was also the case for several ncRNA types, such as lncRNA, miRNA, and scaRNA (*SI Appendix*, Fig. S16*B*). Specifically, we observed that the levels of certain snoRNAs (such as *Snord17*, *Snora23*, and *Snord87*), scaRNAs (such as *Scarna13* and *Scarna6*), lncRNAs (*Platr3*, *Lncenc1*, *Snhg9*, *Gm31659*, and so forth), and miRNAs (*Mir92-2*, *Mir302b*, and *Mir19b-2*) go down (log_2_FC > 1, adjusted *P* < 0.01) after cells exit pluripotency (Fig. 3*B*). In contrast, we also identified that the levels of several lncRNAs (*Tug1*, *Meg3*, *Lockd*) and miRNAs (*Mir298*, *Mir351*, *Mir370*) increase with differentiation (Fig. 3*B*).

Louvain clustering of all collected cells revealed the presence of six molecularly distinct populations, which we assigned to: primed mouse embryonic stem cells (mESC), pre-ectoderm, ectoderm, endoderm, ectomesoderm, and mesoderm (Fig. 3*C*), based on the expression of known lineage-specific marker genes [e.g., *Nanog* and *Pou5f1* for pluripotent cells, *Pax6* and *Olfr787* for ectoderm, *Afp* and *Shh* for endoderm, *Acta2* and *Col3a1* for mesoderm (44)] (*SI Appendix*, Fig. S17 and Dataset S3). The analysis of genes differentially expressed between primed mESCs and each of the identified clusters showed that in addition to well-characterized lineage-specific mRNAs (*SI Appendix*, Fig. S18*A*) (45, 46) and lncRNAs (*Tug1* in ectodermal and *Meg3* mesodermal lineages, respectively) (6), other ncRNA genes—such as miRNAs, scaRNAs, snoRNAs, tRNAs, and histone RNAs—are either specifically expressed or down-regulated within a certain lineage (log_2_FC > 1, adjusted *P* < 0.05) (*SI Appendix*, Fig. S18*B* and Dataset S3).

We next used PAGA (47) to infer a developmental trajectory and compute pseudotime coordinates for each cell in our dataset (*Materials and Methods* and Fig. 3 *D* and *E*). Aligning cells in pseudotime within each lineage further confirmed the existence of expression gradient within different RNA types (Fig. 3*F*). Furthermore, we found that the majority of identified variable noncoding transcripts were germ-layer specific. Examples of such transcripts include *Mir2137*, *Mir320*, *Gm49024*, and *Gm38708* in ectoderm; *Mir351*, *Mir370*, and *Meg3* in mesoderm; as well as *Neat1* in endoderm. *Mir296* and *Mir298* were expressed in both mesoderm and endoderm but were absent in ectoderm (Fig. 3*G*, *SI Appendix*, Fig. S18*B*, and Dataset S3).

Finally, to understand the relationship between mRNA and ncRNA genes, we performed a pairwise correlation analysis of gene expression across all sampled cells. This analysis showed that germ layer-specific miRNAs are correlated with gene sets associated with the proliferation of certain cell lineages (*SI Appendix*, Fig. S19*A* and Dataset S4). For example, miR-370 positively correlated with genes involved in the regulation of nervous system development and miR-351 correlated with genes associated with smooth muscle cell migration and osteoblasts differentiation (*SI Appendix*, Fig. S19). In addition, the expression of ∼50% of identified histone-coding genes correlated with the expression of other protein-coding genes (Spearman’s ρ > 0.5) (*SI Appendix*, Fig. S19). Overall, we found that multiple ncRNAs from all assayed RNA types (e.g., miRNA, snoRNA, snRNA, and so forth) are either positively or negatively correlated with the expression of protein-coding genes (*SI Appendix*, Fig. S19). Most of these ncRNAs represent putative uncharacterized regulators of lineage commitment.

## Discussion

Altogether, Smart-seq-total enables an unbiased exploration of a broad spectrum of coding RNA and ncRNA transcripts in individual cells. Current limitations of Smart-seq-total are: 1) the inability to assay circRNA and 2) the loss of the endogenous polyadenylation status of transcripts. Further modifications to Smart-seq-total can include the selection for a specific transcript length (short vs. long) and depletion of a wider range of overrepresented RNA. We anticipate that Smart-seq-total will facilitate the identification of noncoding regulatory patterns and their functional roles in regulating cellular functions and shaping cellular identity. This could also shift the current protein-centered view of gene regulation toward comprehensive maps featuring both protein and RNA regulators.

## Materials and Methods

### Cell Culture.

HEK293T cells were cultured in complete DMEM high-glucose medium (ThermoFisher, 11965092) supplemented with 5% FBS (ThermoFisher, 16000044), 1 mM sodium pyruvate (ThermoFisher, 11360070), and 100 μg/mL penicillin/streptomycin (ThermoFisher, 15070063). Human primary dermal fibroblasts were obtained from ATCC (PCS-201-012). Cells were cultured and passaged four times in fibroblast basal medium (ATCC, PCS-201-030) supplemented with 5 ng/mL human recombinant FGF (rhFGF) β, 7.5 mM l-glutamine, 50 μg/mL ascorbic acid, 5 μg/mL human i recombinant nsulin, and 1% FBS (Fibroblast Growth kit low serum, ATCC PCS-201-041). MCF7 cells (ATCC, HTB22) were cultured in complete DMEM high-glucose medium (ThermoFisher, 11965092) supplemented with 10% FBS (ThermoFisher, 16000044), 1 mM sodium pyruvate (ThermoFisher, 11360070), and 100 μg/mL penicillin/streptomycin (ThermoFisher, 15070063). Cells were collected 2 to 4 h after passaging, dissociated using 0.25% trypsin-EDTA (ThermoFisher, 25200056) for 2 to 4 min at 37 °C, and sorted in either 96-well plates containing 3 μL lysis buffer or 384-well plates containing 0.3 μL of lysis buffer in each well.

mESCs were maintained and differentiated as described previously (40, 48). Briefly, mESCs were grown in serum-free 2i+LIF medium (complete medium: DMEM/F12 glutaMAX [Gibco, ThermoFisher, 10565018], 1% N2 supplement (Gemini Bio), 2% B27 supplement (Gemini Bio), 0.05% BSA fraction V (ThermoFisher, 15260037), 1% MEM-nonessential amino acids (ThermoFisher, 11140050), and 110 μM 2-mercaptoethanol (Pierce); supplemented with MEK inhibitor PD0325901 (0.8 μM), GSK3β inhibitor CHIR99021 (3.3 μM), and 10 ng/mL mouse LIF (Gibco, PMC9484)] in tissue culture (TC) dishes pretreated with 7.5 μg/mL poly-l-ornithine (Sigma) and 5 μg/mL laminin (BD). To induce spontaneous EB formation, cells were washed with PBS, dissociated with StemPro Accutase (ThermoFisher, A1110501) following the manufacturer’s protocol, transferred to serum-rich medium (complete medium: DMEM/F12 glutaMAX [Gibco], 1% N2 supplement [Gemini Bio], 2% B27 supplement [Gemini Bio], 0.05% BSA fraction V, 1% MEM-nonessential amino acids, and 110 μM 2-mercaptoethanol; supplemented with 10% FBS [ThermoFisher, 10439001]), and diluted to 10^6^ cells/mL. Each 10 μL of cell suspension were plated as a hanging drop in 10 cm^2^ TC dishes (15 to 20 drops per dish). Ten microliters of fresh serum-rich media was added to each drop on the day 4 postseeding. Primed mESCs were collected 6 h after seeding. EBs were collected and dissociated at days 4, 8, and 12 of culture.

### Cell Sort.

Lysis plates were prepared by dispensing 0.3 μL lysis buffer (4 U recombinant RNase inhibitor [RRI; Takara Bio, 2313B], 0.12% TritonX-100 [Sigma, 93443-100ML] in dH_2_O, 1 μM Smart-seq-total oligo-dT primer (5′-Biotin-/5BiosG/CATAGTCTCGTGGGCTCGGAGATGTGTATAAGAGACAGT_30_VN-3′; IDT) in TE buffer (IDTE [10 mM Tris, 0.1 mM EDTA], IDT) (see *SI Appendix*, Table S2 for a full list of oligos used in the present study) into 384-well hard-shell PCR plates (Bio-Rad HSP3901) using Mantis liquid handler (Formulatrix). For the comparison with Smart-seq2 (see *Comparison of Smart-seq2 and Smart-seq-total*, below), 96-well lysis plates were prepared with 3 μL lysis buffer. All plates were sealed with AlumaSeal CS Films (Sigma-Aldrich, Z722634), spun down, and snap-frozen on dry ice.

Cells were stained with calcein-AM and ethidium homodimer-1 (LIVE/DEAD Viability/Cytotoxicity Kit, ThermoFisher, L3224) following the manufacturer’s protocol and individual live cells were sorted in 384-well lysis plates using SONY sorter (SH800S) with a 100-μm nozzle chip. Plates were spun down and stored at −80 °C immediately after sorting.

### Generation of Smart-seq-total v1 Libraries.

To facilitate cell lysis and denaturation of the RNA, 384-well plates were incubated at 72 °C for 3 min, and immediately placed on ice afterward. Next, 0.2 μL of polyA tailing mix, containing 1.25U *E. coli* PolyA (New England Biolabs, M0276S), 1.25× PolyA buffer (New England Biolabs), 1.25 mM ATPs (New England Biolabs) and 4 U of RRI (Takara) were added to each sample. PolyA tailing was carried out for 15 min at 37 °C followed by 72 °C for 30 sec. After polyA tailing plates were immediately placed on ice for 2 to 5 min, 1 μL of reverse-transcription mix, containing 15 U SuperScript II (ThermoFisher), 4 U RRI (Takara), 1.5× First-Strand Buffer, 1.5 μM TSO (Exiqon, 5′-biotin-UCGUCGGCAGCGUCAGUUGUAUCAACUCAGACAUrGrG+G-3′), 7.5 mM DTT, 1.5 M Betaine (Sigma, B0300-5VL), 10 mM MgCl_2_ (Sigma, M1028-10X1ML), and 1.5 mM dNTPs (ThermoFisher, 18427013) was added to each well. Reverse transcription was carried out at 42 °C for 90 min, and terminated by heating at 85 °C for 5 min. Subsequently, 0.3 μL of TSO digestion buffer containing 1 U Uracil-DNA glycosylase (UDG, New England Biolabs, M0280S) were added to each well. Plates were incubated for 30 min at 37 °C. PCR preamplification was performed directly after TSO digestion by adding 3.2 μL of PCR mix to each well, bringing the reaction concentration to 1× KAPA HiFi MIX (Roche), 0.5 μM forward PCR primer (5′-TCGTCGGCAGCGTCAGTTGTATCAACT-3′; IDT), 0.5 μM reverse PCR primer (5′-GTCTCGTGGGCTCGGAGATGTG-3′; IDT). PCR was cycled as follows: 1) 95 °C for 3 min; 2) 21 cycles of 98 °C for 20 s, 67 °C for 15 s, and 72 °C for 6 min; and 3) 72 °C for 5 min. The amplified product was cleaned up using 1× ratio of AMPure beads on Bravo liquid handler platform (Agilent). Concentrations of purified product were measured with a dye-fluorescence assay (Quant-iT PicoGreen dsDNA High Sensitivity kit; Thermo Fisher, Q33120) on a SpectraMax i3x microplate reader (Molecular Devices). Samples were then diluted to 0.2 ng/uL. To generate sequencing libraries, 1.5 μL of diluted samples was amplified in a final volume of 5 μL using 2× KAPA mix and 0.4 μL of 5 μM i5 indexing primer, 0.4 μL of 5 μM i7 indexing primer. PCR amplification was carried out using the following program: 1) 95 °C for 3 min; 2) 8 cycles of 98 °C for 20 s, 65 °C for 15 s, and 72 °C for 4 min; and 3) 72 °C for 5 min.

To perform the library preparation in 96-well plates, we followed the above-described protocol, except that all volumes were scaled up 10 times.

### Generation of Smart-seq-total v2 Libraries.

Smart-seq-total v2 libraries were generated using similar to v1 protocol with a few modifications. Specifically, 0.1 μL of ERCCs (1:300,000 dilution, ThermoFisher) were added to each well, 384-well plates were incubated at 72 °C for 3 min, and immediately placed on ice afterward. Next, 0.2 μL of polyA tailing and 5′-prime capping mix, containing 0.1 U *E. coli* PolyA (New England Biolabs, M0276S), 1.25× PolyA buffer (New England Biolabs), 0.1 mM ATPs (New England Biolabs), 4 U of RRI (Takara), 0.1 U of Vaccina Capping enzyme (New England Biolabs, M2080S), 2.5 nM SAM, and 0.05 mM GTP were added to each sample. PolyA tailing and 5′ prime capping was carried out simultaneously for 15 min at 37 °C followed by 72 °C for 30 sec. After polyA tailing, plates were immediately placed on ice for 2 to 5 min, 1 μL of reverse-transcription mix, containing 15 U SuperScript II (ThermoFisher), 4 U RRI (Takara), 1.5× First-Strand Buffer, 1 μM TSO v2 (Exiqon, 5′-biotin-AdUGGCdUCGGAGAdUGdUGdUAdUAAGAGACAGdUCdUrGrG+G-3′), 7.5 mM DTT, 1.5 M Betaine (Sigma, B0300-5VL), 10 mM MgCl_2_ (Sigma, M1028-10X1ML), and 1.5 mM dNTPs (ThermoFisher, 18427013) was added to each well. Reverse transcription was carried out at 42 °C for 90 min, and terminated by heating at 85 °C for 5 min. Subsequently, 0.3 μL of TSO digestion buffer containing 2 U UDG (New England Biolabs M0280S) were added to each well. Plates were incubated for 60 min at 37 °C. PCR preamplification was performed directly after TSO digestion by adding 3.2 μL of PCR mix to each well, bringing the reaction concentration to 1× KAPA HiFi MIX (Roche), 0.5 μM forward PCR primer (5′-GCTCGGAGATGTGTATAAGAGACAG-3′; IDT), 0.5 μM reverse PCR primer (5′- TCGTCGGCAGCGTCAGTTG-3′; IDT). PCR was cycled as follows: 1) 95 °C for 3 min; 2) 21 cycles of 98 °C for 20 s, 65 °C for 15 s, and 72 °C for 5 min; and 3) 72 °C for 5 min. The amplified product was cleaned up using 1.8× ratio of AMPure beads on Bravo liquid handler platform (Agilent). Concentrations of purified product were measured with a dye-fluorescence assay (Quant-iT PicoGreen dsDNA High Sensitivity kit; Thermo Fisher, Q33120) on a SpectraMax i3x microplate reader (Molecular Devices). Samples were then diluted to 0.2 ng/uL.

To generate sequencing libraries through direct indexing, 1.5 μL of diluted samples was amplified in a final volume of 4 μL using 2× KAPA mix, 0.025 μL of 1 μM Smart-seq-total index Amp primer (5′- GTCTCGTGGGCTCGGAGATGTGTATAAGAGACAGTC-3′), and 0.4 μL of 5 μM i5 indexing primer, 0.4 μL of 5 μM i7 indexing primer. PCR amplification was carried out using the following program: 1) 95 °C for 3 min; 2) 10 cycles of 98 °C for 20 s, 62 °C for 15 s, and 72 °C for 5 min; and 3) 72 °C for 5 min.

To generate libraries through tagmentation, we followed previously described procedure (49).

### Library Pooling, Ribosomal Sequence Digestion, and Sequencing.

After library preparation, wells of each library plate were pooled using a Mosquito liquid handler (TTP Labtech). If pooling tagmented and nontagmented libraries, the two types of libraries were mixed in ∼1:1 molar ratio. Pooling was followed by a purification with 0.8× AMPure beads (Fisher, A63881). Ribosomal reads were digested using DASH (depletion of abundant sequences by hybridization), as described previously (23) (see *SI Appendix*, *Supplementary Protocol* for details). Briefly, the guides designed to target 45S rRNA and other abundant sequences (*SI Appendix*, Table S1) were combined with tracer RNA and assembled with Cas9 protein in 2:1 ratio. The assembled complexes were incubated with the sequencing library in 1× Cas9 buffer (*SI Appendix*, Table S1) for 1 h at 37 °C. Following rRNA sequence digestion, Cas9 was inactivated through incubation with proteinase K for 15 min at 50 °C. The library was then purified twice, first using 1.2× AMPure beads to DNA ratio. then using 2% Pippin Prep gels (Sage Sciences) in the 210- to 600-bp range.

Library quality was assessed using capillary electrophoresis on a Fragment Analyzer (AATI), and libraries were quantified by qPCR (Kapa Biosystems, KK4923) on a CFX96 Touch Real-Time PCR Detection System (Bio-Rad). Plate pools were normalized to 2 nM and equal volumes from eight plates were mixed together to make the sequencing sample pool. A PhiX control library was spiked in at 10% before sequencing. Smart-seq-total v1 libraries were sequenced on the NovaSEq. 6000 Sequencing System (Illumina) using 1 × 75- or 1 × 100-bp single-end reads (using custom Read 1 sequencing primer: 5′- TCGGCAGCGTCAGTTGTATCAACTCAGACATGGG-3′) and 2 × 12-bp index reads. Smart-seq-total v2 libraries were sequenced on the NextSEq. 500 Sequencing System (Illumina) using 1 × 30- and 1 × 112-bp paired-end reads (using custom Read 1 sequencing primer: 5′-TCGTCGGCAGCGTCAGTTGTATCAACTCAGAC-3′) and 2 × 12-bp index reads (using custom Index 2 sequencing primer: 5′- GTCTGAGTTGATACAACTGACGCTGCCGACGA-3′).

### Data Processing.

Sequences from the NovaSeq were de-multiplexed using bcl2fastq v2.19.0.316. The analysis of Smart-seq-total v1 and Smart-seq-total v2 data were carried out as described in GitHub Smart-seq-total page (https://github.com/aisakova/smart-seq-total/blob/master/README.md). Briefly, for Smart-seq-total v1, reads were trimmed from polyA tails using cutadapt v1.18 with the following parameters: -m 18 -j 4 -a AAAAAAAAAA -a TTTTTTTTTT. Reads were then aligned to the human (GRCh38) or mouse (GRCm38) genomes using STAR_v2.7.0d (50) with the following parameters: –outFilterMismatchNoverLmax 0.05 –outFilterMatchNmin 18 –outFilterMatchNminOverLread 0 –outFilterScoreMinOverLread 0 –outMultimapperOrder Random. Reads mapping to multiple locations were assigned either to a location with the best mapping score or, in the case of equal multimapping score, to the genomic location randomly chosen as “primary.”

Transcripts were counted using *featureCounts* v1.6.1 (51) with the following parameters: -M –primary -s 1. GENCODE v32 and GENCODE M23 (52) annotations were used for human and mouse reads, respectively. tRNA was quantified using high-confidence gene set obtained from GtRNA (53). To account for multimappers, “primary” alignment reported by STAR was counted. For miRNA and tRNA, all reads mapping to arms or the stem loop were summed to quantify the expression at the gene level.

### Comparison of Smart-seq2 and Smart-seq-total.

HEK293T cells were sorted in 96-well plates containing 3 μL of lysis buffer (as described above). The reaction volumes for Smart-seq-total were scaled up 10 times compared to 384-plate format (i.e., RNA from each cell was polyadenylated in 5 μL, reverse-transcribed in 15 μL, and cDNA was preamplified in 15 μL total volume). We retrieved Smart-seq2 data from Picelli et al. (19) (GSE49321). Smart-seq2 and Smart-seq-total reads were mapped using STAR and counted using featureCounts, as described above. Comparisons between protocols in *SI Appendix*, Fig. S1*C* were generated on depth-normalized libraries, using 2.5 million randomly selected reads per adaptor-trimmed library (or all reads for libraries that had less than 2.5 million reads) to compute expression levels (counts per million, cpm).

### Unsupervised Clustering and Dimensionality Reduction Analysis of Human Cell Types.

Standard procedures for filtering, variable gene selection, dimensionality reduction, and clustering were performed using the Seurat package v3.1.4 (54). Cells with fewer than 2,000 detected genes and those with more than two Mio reads were excluded from the analysis. Counts were log-normalized for each cell using the natural logarithm of 1 + cpm. Variable genes were selected based on overdispersion analysis and projected onto a low-dimensional subspace using PCA. The number of PCs was selected on the basis of inspection of the plot of variance explained. Cells were visualized using a two-dimensional t-SNE of the PC-projected data. Dimensionality reduction parameters for t-SNE (resolution and number of PCs) were adjusted on a per cell type and per biotype basis and can be viewed in the Rmd files available on GitHub. Cells were assigned a cell cycle score using Seurat’s CellCycleScoring() function using cell cycle markers described in Tirosh et al. (30).

### Clustering of Coding and Noncoding Genes.

Clusters of coding and noncoding genes shown in Fig. 2*B* were computed and visualized using the DEGreport R package (55). The top 250 marker genes for each cell-cycle phase and all noncoding genes with average expression ln(cpm+1) > 0.05 in at least one phase were used for this analysis. Gene-expression values were normalized using variance stabilizing transformation (56) before clustering. Further details of the analysis can be viewed in the Rmd files available on GitHub.

### Correlation between Mature miRNAs and mRNA.

Total RNA fraction and a corresponding small RNA fraction of a Smart-seq-total v2 library were sequenced separately on NextSeq 500 sequencer. Sequencing data from both libraries was processed as described above (also see https://github.com/aisakova/smart-seq-total/blob/master/README.md). Small RNA and total RNA count tables were normalized and log-transformed separately. Pearson coefficients were computed for all pair-wise correlations between miRNAs and all other genes detected in ∼300 HEK293T cells [present in at least 30 cells, ln(cpm+1) > 1]. Predicted and validated targets were annotated using *multiMiR* pipeline (scanning predictions from various databases, such as TargetScan, mirTarBase, miRanda, and so forth) (57). Predicted targets were limited to those listed in two or more external databases. Validated targets with “weak” evidence were excluded from the analysis [(abs(*r*)) >0.5, adjusted *P* value <0.01] (*SI Appendix*, Fig. S15*A*). GO was performed on ∼100 to 200 top correlated (*r* > 0.3, adjusted *P* < 0.01) or ∼100 anticorrelated (*r* < −0.3, adjusted *P* < 0.01) genes. The 10 GO terms with lowest *P* value in Kolmogorov–Smirnov test were used (*SI Appendix*, Fig. S15*B*)

### Preprocessing and Clustering of mESCs.

Standard procedures for filtering, variable gene selection, dimensionality reduction, and clustering were performed using the Seurat package v3.1.4 (54). Cells with fewer than 1,000 detected genes and those with more than two Mio reads were excluded from the analysis. Counts were log-normalized for each cell using log_1_p(counts) and 1e4 scale factor. Variable genes were projected onto a low-dimensional subspace using PC analysis. The number of PCs was selected on the basis of inspection of the variance explained plot. A shared nearest-neighbor graph was constructed on the basis of the Euclidean distance in the low-dimensional subspace spanned by the top PCs. Cells were visualized using the uniform manifold approximation and projection (UMAP) algorithm (58) of the PC-projected data. Clusters were annotated based on the expression of known marker genes corresponding to one of the three germ layers. Cells were assigned a cell-cycle score using Seurat’s CellCycleScoring() function and cell-cycle markers described in Tirosh et al. (30)

### Developmental Trajectory Inference of EB Differentiation.

Developmental trajectory of mESC differentiation was inferred using PAGA through dynoverse wrapper (59). Pseudotime coordinates computed from the trajectory were appended to Seurat object and further used to generate Fig. 3 *C*–*F*.

### Correlation between Coding and Noncoding RNA Levels.

Spearman coefficients were computed for all pair-wise correlations of expressed genes [average ln(cpm+1) > 2 across all cells]. The resulting matrix was subset to only mRNA:ncRNA correlations. Pairs with Spearman’s ρ > 0.5 were used to generate a chord diagram shown in *SI Appendix*, Fig. S17*A* and pairs with Spearman’s ρ < −0.5 were used to generate a chord diagram shown in *SI Appendix*, Fig. S17*B*.

## Supplementary Material

Supplementary File

Supplementary File

Supplementary File

Supplementary File

Supplementary File

## Data Availability

The data reported in this paper have been deposited in the Gene Expression Omnibus (GEO) database, https://www.ncbi.nlm.nih.gov/geo (accession no. GSE151334) (61). All code used for analysis is available on GitHub (https://github.com/aisakova/smart-seq-total/).

## Change History

January 21, 2022: The byline has been updated.
