# Supplementary material for: Single-cell quantification of a broad RNA spectrum reveals unique noncoding patterns associated with cell types and states
Source: Proc Natl Acad Sci U S A. 2021 Dec 15;118(51):e2113568118. doi: 10.1073/pnas.2113568118 (PMC8713755; doi:10.1073/pnas.2113568118)
Supplement: Supplementary File [file pnas.2113568118.sapp.pdf]

PNAS
www.pnas.org

Supplementary Information for:

Single cell profiling of total RNA using Smart-seq-total

Alina Isakova[1], Norma Neff [3], Stephen R. Quake[1,2,3]

[1]Department of Bioengineering, Stanford University, Stanford, California, USA
[2]Department of Applied Physics, Stanford University, Stanford, California, USA
[3]Chan Zuckerberg Biohub, San Francisco, California, USA

Correspondence
steve@quake-lab.org

This PDF file includes:

- Supplementary text
- Figures S1 to S19
- Tables S1 to S3
- SI References

Other supplementary materials for this manuscript include the following:

- Datasets S1 to S7

Supplementary text(protocol):

CRISPR-mediated depletion of unwanted molecules from Smart-seq-total libraries (DASH[1]).

Supplementary figures:

Supplementary figure S1. Smart-seq-total performance.
Supplementary figure S2. Smart-seq-total v2 workflow.
Supplementary figure S3. Mapping statistics and gene coverage.
Supplementary figure S4. Comparison of Smart-seq-total with other scRNA-seq methods.
Supplementary figure S5. Number of coding and non-coding genes detected in primary fibroblasts, HEK293T and MCF7 cells.
Supplementary figure S6. Most abundant transcripts detected in primary fibroblasts, HEK293T and MCF7 cells.
Supplementary figure S7. Depletion of abundant miscRNA.
Supplementary figure S8. Cell type-specific marker genes grouped by RNA type.
Supplementary figure S9. Transcripts dynamically changing over the cell cycle identified in primary fibroblasts.
Supplementary figure S10. Transcripts dynamically changing over the cell cycle identified in HEK293T cells.
Supplementary figure S11. Transcripts dynamically changing over the cell cycle identified in MCF7 cells.
Supplementary figure S12. Clusters of coding and non-coding genes dynamically expressed throughout the cell cycle.
Supplementary figure S13. Cell cycle bias in cell clustering.
Supplementary figure S14. Sequencing of small RNA fraction of Smart-seq-total libraries.
Supplementary figure S15. Correlation of miRNA and mRNA levels across individual HEK 293T cells.
Supplementary figure S16. Number of coding and non-coding genes detected in mESCs at each stage of EB formation.
Supplementary figure S17. UMAP plots of lineages and key marker genes.
Supplementary figure S18. Genes differentially expressed between lineages.
Supplementary figure S19. Coding and non-coding gene correlation analysis.

Supplementary tables:

Supplementary table S1. CRISPR guides for rRNA depletion.
Supplementary table S2. Smart-seq-total primers used in the present study.
Supplementary table S3. Smart-seq-total cost estimate.

Supplementary datasets:

Supplementary Dataset S1. Cell cycle-specific gene clusters.
Supplementary Dataset S2. miRNA-mRNA target correlation scores.
Supplementary Dataset S3. Coding and non-coding markers of germ layer specification.
Supplementary Dataset S4. Correlation of germ layer-specific miRNAs and other coding and non-coding genes.
Supplementary Dataset S5. miRNA count tables obtained for 24 HEK293T cells.
Supplementary Dataset S6 and S7. Matching total and small RNA count tables obtained from 301 HEK293T cells.

Supplementary protocol

Prepare the guide RNA

1. Resuspend each crRNA oligo in IDTE buffer to a stock concentration of 100 µM (add 20uL of buffer to each well).
2. Make a pool of all 57 crRNA: pool 2 uL from guide 1 to 31 (A1 to C7, noA12) and 1uL from guides 32:57 (D1 to F2) to obtain 88 uL (each guide will be at ~2.3 uM).
3. Resuspend each tracrRNA in IDTE buffer to a stock concentration of 100 µM.
4. Mix the crRNA and tracrRNA oligos in equimolar concentrations in a sterile microcentrifuge tube to a final duplex concentration of 10 µM.

Volume	**Component**
88 uL	100 µM Alt-R CRISPR-Cas9 crRNA
88 uL	100 µM Alt-R CRISPR-Cas9 tracrRNA
704 uL	Nuclease-Free Duplex Buffer
1140 uL	**Total volume**

4. Heat the duplex at 95°C for 5 min.
5. Remove from heat and allow to cool to room temperature (15–25°C). Store as 5-10uL aliquots at -80C for limited time.

Create the RNP complex

6. Combine the guide RNA and Cas9 enzyme (20uM stock from NEB) in equimolar amounts:

Volume	**Component**
28 uL	10 µM crRNA:tracrRNA
14 uL	S.p. Cas9 (20uM)
238 uL	PBS
280 uL	**Total volume**

† Cas9 RNP complexes can be made in PBS or in Cas9 dilution buffer (30 mM HEPES, 150 mM KCl, pH 7.5).

7. Incubate at room temperature for 5–10 min for optimal formation of the RNP complex.

Perform the *in vitro* digestion reaction

8. Assemble the reaction at room temperature (15–25°C):

Volume	**Component**
40 uL	10X Cas9 Nuclease Reaction Buffer
280 uL	1uM Cas9 RNP
80 uL	Sequencing library (10 nM) (Use nuclease free water to dilute if needed)
400 uL	**Total volume**

† The molar ratio of Cas9 RNP:Library should be 40:1 or higher. Final concentration of RNPs is 100nM (2.3 nM for each guide).

9. Incubate the reaction at 37°C for 60 min.
10. Add 1 µL Proteinase K (20 mg/mL) to the reaction, then incubate the mixture at 56°C for 10 min to release the DNA substrate from the Cas9 endonuclease.
11. Clean-up using 0.8X or 1X ratio of SPRI Select. Elute in 30uL.

**Cytoplasmic rRNA and miscRNA**	**Target Sequence**	
RNA7SK_1	GTCCATTTGTAGGAGAACGT	A01
RNA7SK_2	TCCAAATGAGGCGCTGCATG	A02
RNA7SK_3	ACCCTACGTTCTCCTACAAA	A03
RNA7SK2_1	CACTAAGTTCGGCATCAATA	A04
RNA7SK2_2	GACCACCAGGTTGCCTAAGG	A05
RNA7SK2_3	*GGAGTGCAGTGGCTATTCAC*	
7SL_1	CACTAAGTTCGGCATCAATA	A06
7SL_2	GACCACCAGGTTGCCTAAGG	A07
5.8S_1	CGACACTTCGAACGCACTTG	A08
5.8S_2	ACTCTTAGCGGTGGATCACT	A09
5S.1	TCTGATCTCGGAAGCTAAGC	A10
5S.2	CTCCCATCCAAGTACTAACC	A11
**mtRNA**		
MT-RNR1_1	TTGACCTAACGTCTTTACGT	B01
MT-RNR1_2	CACGAAATTGACCAACCCTG	B02
MT-RNR1_3	AGGGTGAACTCACTGGAACG	B03
MT-RNR1_4	AGTAGAGTGCTTAGTTGAAC	B04
MT-RNR1_5	GTAGTCTATTTTGTGTCAAC	B05
MT-RNR1_6	AGAGGAGACAAGTCGTAACA	B06
MT-RNR1_7	GGGTTTATCGATTACAGAAC	B07
MT-RNR2_1	CTGAACTCCTCACACCCAAT	B08
MT-RNR2_2	AAGGTTGTCTGGTAGTAAGG	B09
MT-RNR2_3	CCTCACTGTCAACCCAACAC	B10
MT-RNR2_4	GCCGTTAAACATGTGTCACT	B11
MT-RNR2_5	GCTGCTTTTAGGCCTACTAT	B12
MT-RNR2_6	TTGGACAACCAGCTATCACC	C01
MT-RNR2_7	CTGTTCTTAGGTAGCTCGTC	C02
MT-RNR2_8	TGAGATGATATCATTTACGG	C03
MT-RNR2_9	TGTTCCGTTGGTCAAGTTAT	C04
MT-RNR2_10	TTTGGTAGTTTAGGACCTGT	C05
MT-RNR2_11	CTGTATGAATGGCTCCACGA	C06

MT-RNR2_12	TTTAATAGCGGCTGCACCAT	C07
**18S and 28S**		
18S_1	TAGAGTCACCAAAGCCGCCG	D01
18S_2	CTCCAATGGATCCTCGTTAA	D02
18S_3	TAGAGCTAATACATGCCGAC	D03
18S_4	ACGGCTACCACATCCAAGGA	D04
18S_5	AATTACCCACTCCCGACCCG	D05
18S_6	CATGTCTAAGTACGCACGGC	D06
18S_7	CATGGTGACCACGGGTGACG	D07
18S_8	ACTCAGCTAAGAGCATCGAG	D08
18S_9	AGTCGTAACAAGGTTTCCGT	D09
18S_10	CATCCAATCGGTAGTAGCGA	D10
18S_11	CATCACGAATGGGGTTCAAC	D11
18S_12	GTTGAGTCAAATTAAGCCGC	D12
18S_13	ATTCTTGGACCGGCGCAAGA	E01
28S_1	CGGACAAACCCTTGTGTCGA	E02
28S_2	GGACGAAGGGCACTCCGCAC	E03
28S_3	CGGTTCCTCTCGTACTGAGC	E04
28S_4	CACTAATAGGGAACGTGAGC	E05
28S_5	ATTCGTAGACGACCTGCTTC	E06
28S_6	GGCTCCCAAACCACGCTCCC	E07
28S_7	GGTCGACCCGGGACACGTGC	E08
28S_8	CCGTCGGGACGAACCGCAAC	E09
28S_9	CTCGTGACCTCTCCTCGGTC	E10
28S_10	CCGTCTCGGTCGGCACTCCG	E11
28S_11	CTAAGTGTCAAGAGCCGAGA	E12
28S_12	GAGGAAGCATTACTGGCTGA	F01
28S_13	ACGGTGGAGCTGGGACCACG	F02

Supplementary figure captions:

Supplementary figure S1. Smart-seq-total performance.

a. Sequencing scheme of a scRNA-seq library prepared with Smart-seq-total.

b. Bioanalyzer traces showing fragment sizes of amplified cDNA prepared from single cells using Smart-seq-total with and without TSO removal step.

c. Average number of genes per biotype detected by Smart-seq2 and Smart-seq-total in single HEK293T cells. Smart-seq-total libraries were obtained from cells sorted in 3 uL of lysis buffer (See *Methods* **Comparison of Smart-seq2 and Smart-seq-total** for details). Genes were assigned to a specific biotype based on GENCODE v32 annotation for the reference genome. tRNA was quantified using high-confidence gene set obtained from GtRNAdb. Adaptor-depleted libraries, for both Smart-seq2 and Smart-seq-total, were depth normalized to ~2.5 Mio reads per cell. Error bars denote standard deviation (n=7).

d. Mean number of genes per biotype detected by Smart-seq2 and Smart-seq-total in single HEK293T cells. Same as **c** but for tRNA, miscRNA, TEC, intronic and antisense transcripts.

e. Read coverage of multiple gene biotypes shown for Smart-seq2 and Smart-seq-total data. Computed as a sum of n=7 cells.

Supplementary figure S2. Smart-seq-total v2 workflow.

a. Sequencing scheme of a scRNA-seq library prepared with Smart-seq-total v2.

b. Schematic workflow of Smart-seq-total v2. Following cell lysis, total cellular RNA is polyadenylated and 5'prime capped, primed with anchored UMI-containing oligo dT and reverse transcribed in a presence of the custom degradable TSO. After reverse transcription, TSO is enzymatically cleaved, single-stranded cDNA is amplified and cleaned up. Amplified cDNA is then either tagmented or directly indexed, pooled and depleted from ribosomal sequences using DASH[1]. Resulting indexed libraries are then pooled and sequenced on Illumina platform.

c. Effects of the 5'-prime capping on the cDNA yield.

d. Fragment analyzer traces showing the effect of the reverse primer addition during the cDNA amplification step. Left-over UMI-containing oligodT (dT22VN) from the RT step remains in the reaction during the cDNA amplification thus could potentially prime cDNA and 'inflate' the UMI count. However, the melting temperature of Smart-seq (v2) oligodT is designed to be > 10 degrees below the annealing temperature of our PCR primers. Under these conditions and in the absence of reverse prime, the oligodT yields negligible amplification of cDNA.

e. Schematic diagram of Smart-seq-total v2 data analysis.

f. Correlation between raw and UMI-corrected counts obtained from Smart-seq-total v2 scRNA-seq data generated from 39 HEK293T cells. Each dot represents individual gene in a given cell.

Supplementary figure S3. Mapping statistics and gene coverage.

a. Schematic view of the library preparation workflow following cDNA amplification in Smart-seq-total v1. Gene body coverage, computed as an average across housekeeping genes using RSeQC v4.0[2]. Each colored line represents an individual cell.
b. Same as **a**, but for Smart-seq-total v2.
c. Mapping statistics generated based on data generated from the workflow described in **a**.
d. Same as **c**, but for Smart-seq-total v2.
e. Absolute number of UMIs detected per input ERCC molecules. Lines indicate the range found across wells. The data has not been depth normalized.
f. Sensitivity of Smart-seq-total v2, calculated as the number of ERCC molecules needed to reach a 50% detection probability as described in Svensson et al.[3] and Bagnoli et al.[4] is equal to 4.6 molecules (median across 39 samples). Per-cell distributions are shown using violin plots with vertical lines and numbers indicating the median across wells.

Supplementary figure S4. Comparison of Smart-seq-total with other scRNA-seq methods.
a. Qualitative comparison of Smart-seq-total, Smart-seq2/3, Holo-seq, SUPeR-seq and MATQ-seq.
b. Number of genes detected withing each RNA type across different methods (cpm>=1). Datasets were depth normalized to the lowest number of reads per library (~1 Mio reads per cell). Error bars denote standard deviation. Numbers in the legend indicate the number of cells included in the comparison (available from the respective studies).
c. Gene body coverage, computed as an average across all genes using RSeQC v4.0[2]. Coverage for Smart-seq-total tagmented and non-tagmented libraries is computed separately.
d. Fraction of reads mapping to exons, introns and intergenic regions across compared methods.

Supplementary figure S5. Number of coding and non-coding genes detected in primary fibroblasts, HEK293T and MCF7 cells.
a. Number of counts and number of genes per cell grouped by RNA type. Computed based on 612 profiled cells.
b. Number of genes, number of counts as well as the percentage of mitochondrial and histone RNA per cell computed for primary fibroblasts, HEK293T and MCF7 cells.
c. Number of detected genes by RNA type in the three profiled cell types.

Supplementary figure S6. Most abundant transcripts detected in primary fibroblasts, HEK293T and MCF7 cells.
Top 100 genes in each of the three profiled cell types supported by the largest number of reads. Red arrows indicate cell type-specific genes.

Supplementary figure S7. Depletion of abundant miscRNA**.**

a. Distribution of mapped reads across RNA types in HEK293T cells and MCF7 cells**.** The abundant RNA species were depleted from the final libraries using either v1 or v2 sets of CRISPR guides (DASH v1 or DASH v2 respectively).

b. Levels of ACTB, RN7SK and RN7SL detected in libraries depleted using either DASH v1 or DASH v2.

Supplementary figure S8. Cell type-specific marker genes grouped by RNA type.

a. Dot plots of marker genes identified in primary fibroblasts, HEK293T and MCF7 cells grouped by RNA type.

b. Relative levels of 21 tRNA (iMet-tRNA) types identified in fibroblasts, HEK293T and MCF7 cells as a fraction of total reads mapped to tRNA genes.

c. Loadings of the first ten principal components that define the separation of primary fibroblasts, HEK293T and MCF7 based on 'other small ncRNA' (snoRNA, scaRNA, snRNA and tRNA) shown in Figure 1f (left panel).

d. Top 25 small ncRNAs (among snoRNA, scaRNA, snRNA and tRNA classes) differentially expressed across the cell types.

Supplementary figure S9. Transcripts dynamically changing over the cell cycle identified in primary fibroblasts.

Coding and non-coding RNAs differentially expressed across the cell cycle in primary dermal fibroblasts. Grouped by RNA type. Circular charts depict average expression of a given gene across all cells identified to be in a certain phase of the cell cycle.

Supplementary figure S10. Transcripts dynamically changing over the cell cycle identified in HEK293T cells.

Same as Supplementary fig. 9 but for HEK293T cells.

Supplementary figure S11. Transcripts dynamically changing over the cell cycle identified in MCF7 cells.

Same as Supplementary fig. 9-10 but for MCF7 cells.

Supplementary figure S12. Clusters of coding and non-coding genes dynamically expressed throughout the cell cycle.

Universal and cell-type specific gene clusters composed of coding and non-coding genes that dynamically change through the cell cycle. Hierarchical clustering was performed on a mixed-gene group composed of top 750 mRNAs differentially expressed through the cell-cycle and non-coding

genes expressed in least one phase. For more information on cluster composition see Supplementary table 2.

Supplementary figure S13. Cell cycle bias in cell clustering.

a. t-SNE plot of the three profiled human cell types generated based on all detected genes.

b. t-SNE plot of the three profiled human cell types colored by cell cycle phase.

c. t-SNE plots colored by expression of selected cell-cycle and histone genes. Color scale adjacent to each plot denotes the expression of a given gene as ln(cpm+1).

Supplementary figure S14. Sequencing of small RNA fraction of Smart-seq-total libraries.

a. Mapping statistics of the obtained small RNA libraries.

b. Coverage of small RNAs detected in HEK293T cells across the length of the precursor RNAs (divided into 100 bins).

c. Overlap of miRNAs detected by Smart-seq-total v2 and in Faridani et al.[5] in single HEK293T cells as well in the bulk RNA sample.

d. Correlation of selected mature miRNA expression across 24 HEK 293T cells. Schematics of miR-17/92and its paralog miRNA clusters is depicted in the upper left corner.

e. Levels of the -5p and -3p arms of the same miRNA detected in HEK293T cells. Average values across 24 cells are shown. Error bars denote standard deviation.

f. Correlation of miR-92a-3p and miR-25-3p, which belong to the same miRNA family, levels across 301 HEK293T cells.

Supplementary figure S15. Correlation of miRNA and mRNA levels across individual HEK 293T cells.

a. Pair-wise correlation scores computed for miR-92a-3p and miR-25-3p and the rest of the genes (~8'000 genes; each gene present in at least 30 cells at cpm>1) detected in individual HEK293T cells. P-values of correlations were computed using t-test and adjusted using Benjamini-Hochberg correction.

b. Top 10 enriched GO terms computed based on the most correlated and anti-correlated with miR-92a-3p (left) and miR-25-3p (right) coding genes.

Supplementary figure S16. Number of coding and non-coding genes detected in mESCs at each stage of EB formation.

a. Total number of detected genes and counts, as well as the percentage of mitochondrial and histone RNA, detected in each cell collected at day 0, day4, day 8 and day 12 of embryoid body formation.

b. Number of counts and number of genes detected in each cell at days 0, 4, 8 and 12 of embryoid body formation. Grouped by RNA type.

Supplementary figure S17. UMAP plots of lineages and key marker genes.

a. UMAP plot of collected murine cells colored by annotated cell types.

b. UMAP plots showing the expression of lineage markers. Color scale adjacent to each plot denotes the expression of a given gene as ln(normalized counts +1).

c. UMAP plot of collected murine cells colored by cell cycle phase.

Supplementary figure S18. Genes differentially expressed between lineages.

a. Genes differentially expressed between annotated lineages and primed mESCs.

b. Non-coding genes differentially expressed between annotated lineages and primed mESCs. Primed mESCs are marked by the expression of *Nanog, Pou5f1* and *Esrrb*.

c. UMAP plots showing the expression of miRNAs in profiled cells. Color scale adjacent to each plot denotes the expression of a given gene as ln(normalized counts +1).

Supplementary figure S19. Coding and non-coding gene correlation analysis.

a. Pair-wise correlation scores computed for germ layer specific miRNAs and the rest of the genes detected across individual differentiating cells. Average normalized expression of each gene is computed across all cells. Correlations with Pearson's r >0.3 and <-0.3 and Benjamini-Hochberg adjusted P-value <0.01 are shown. Top 10 enriched GO terms computed based on the most correlated and anti-correlated genes.

b. Positively correlated coding and non-coding genes in differentiating mESCs. Gene correlation was evaluated through pairwise correlation analysis across all cells collected at four stages of EB formation. Genes with spearman rho > 0.5 (P-value <10^-5) were considered co-expressed.

c. Anticorrelation of coding and non-coding genes. Evaluated through pairwise gene correlation analysis across all cells. Genes with spearman rho < -0.5 were considered to be anticorrelated (P-value <0.01).

Supplementary figure 1

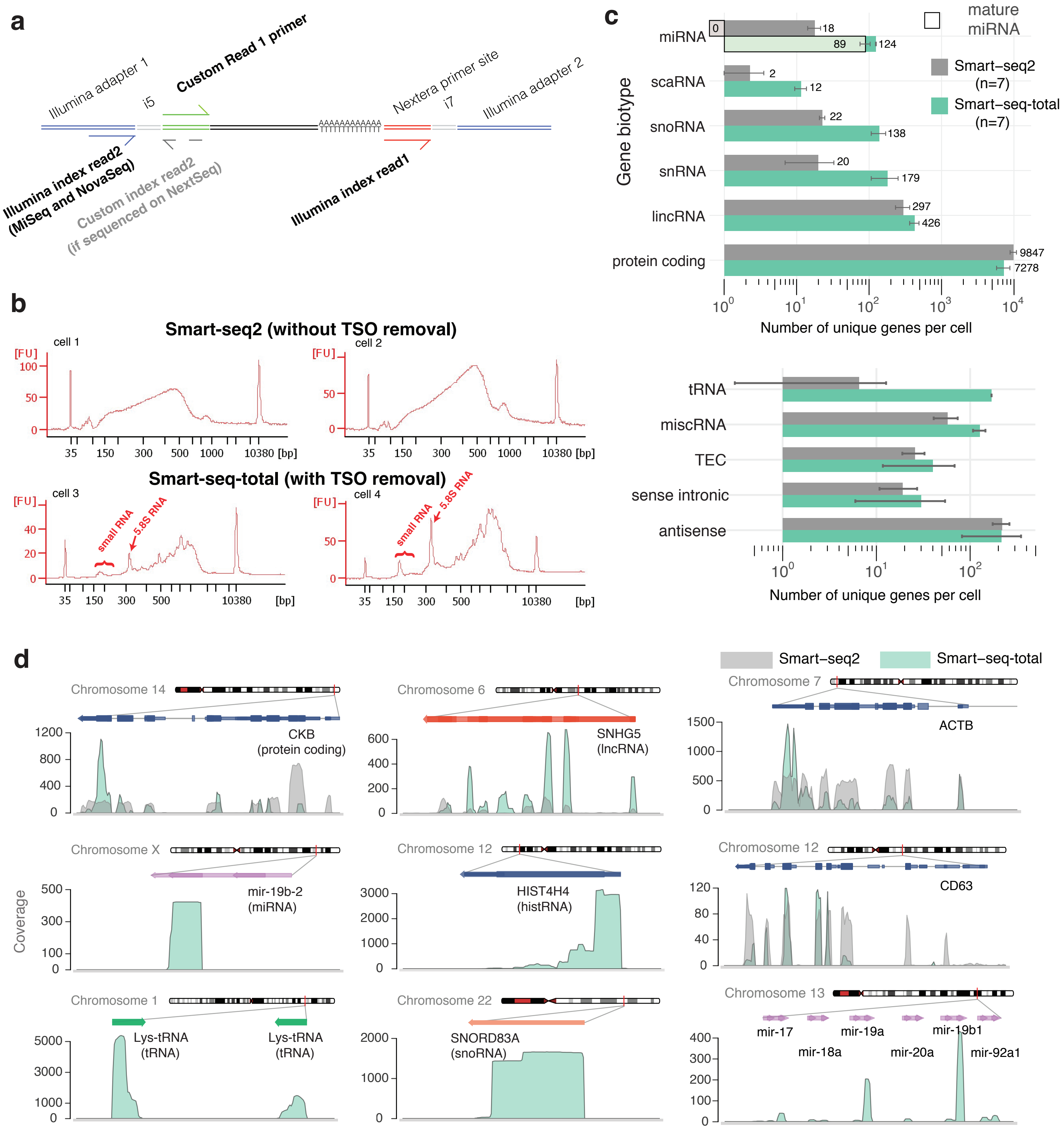

Supplementary figure 2

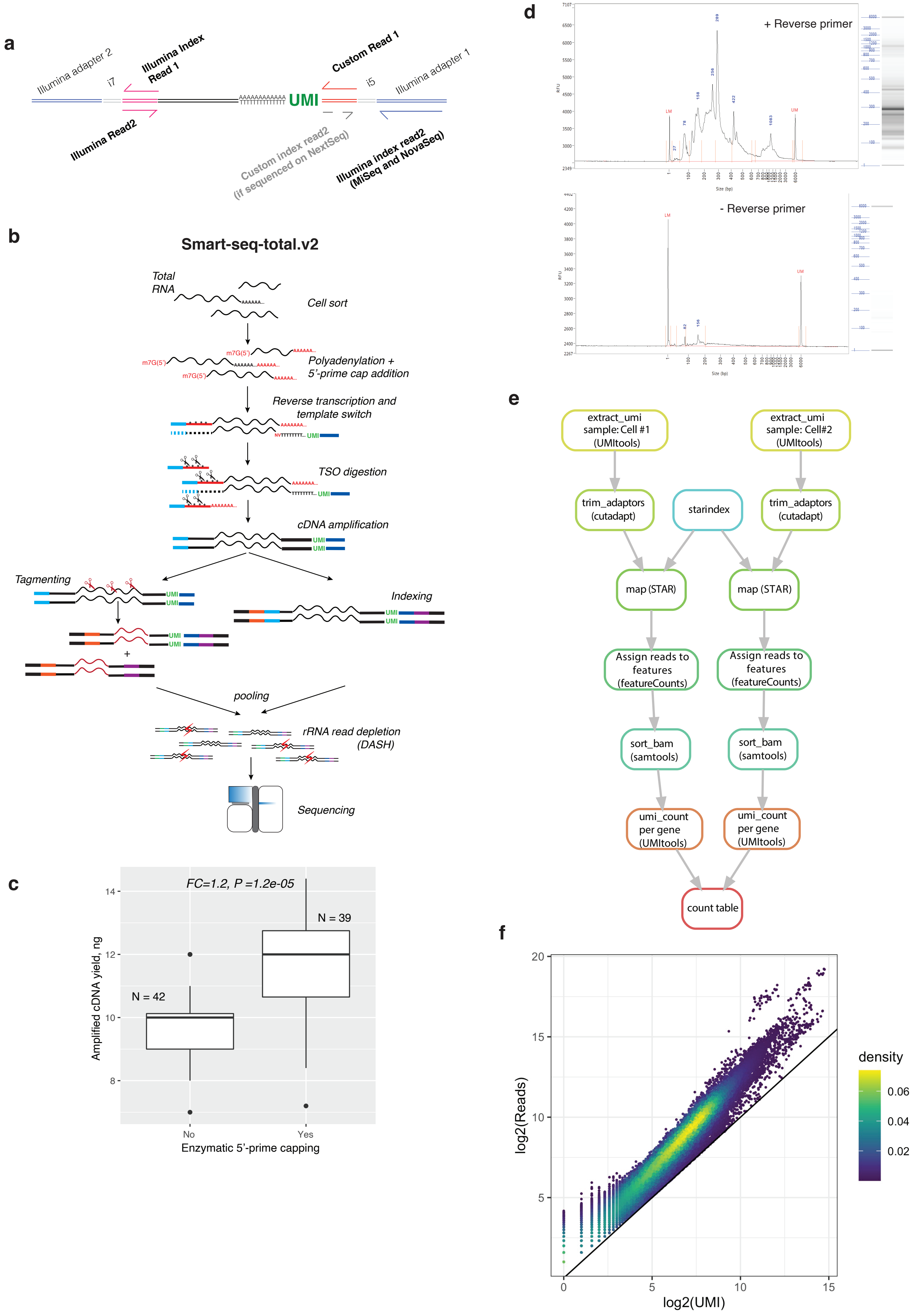

Supplementary figure 3

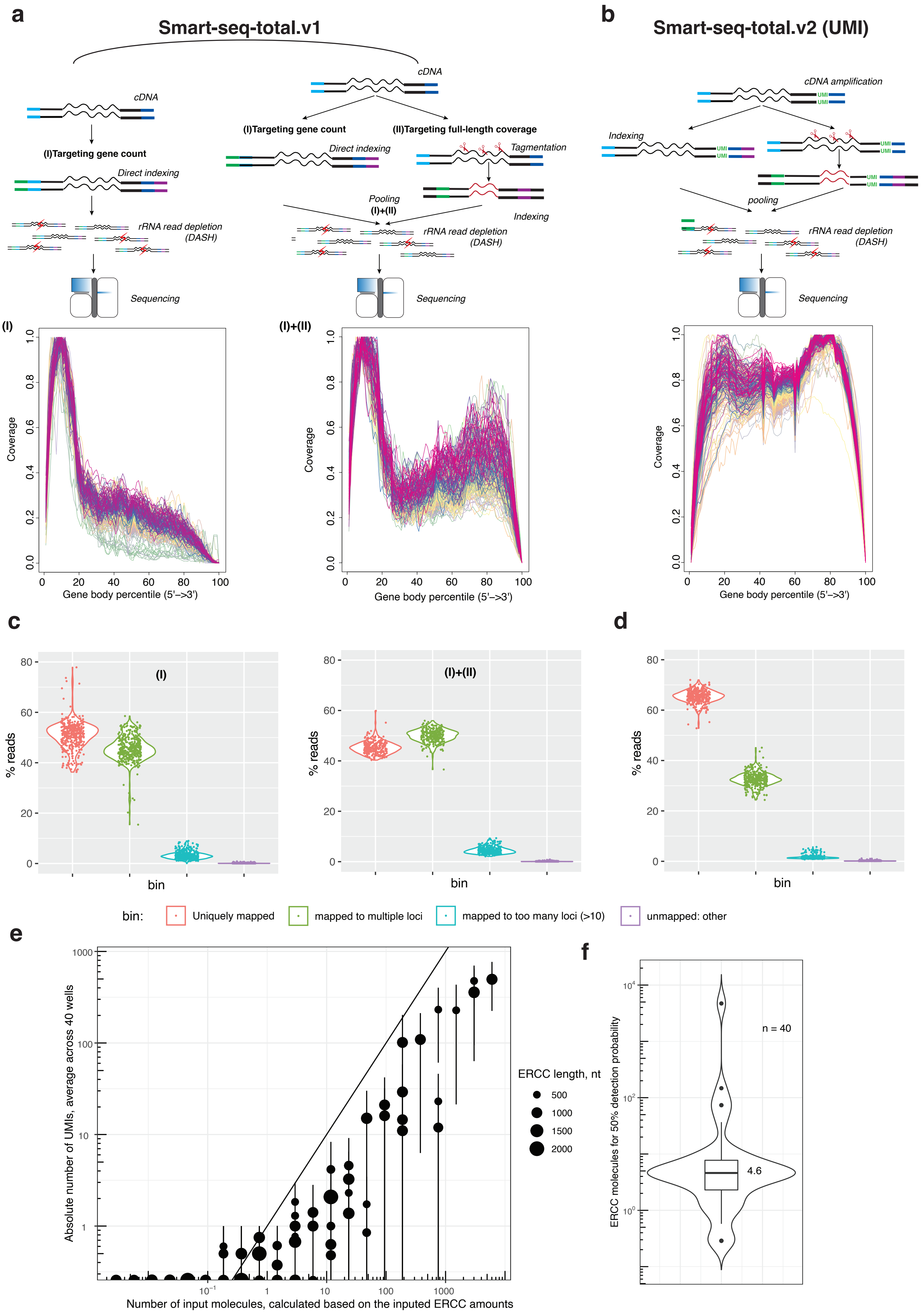

Supplementary figure 4

a

	Smart-seq-total	Smart-seq2/3	Holo-seq	SUPeR-seq	MATQ-seq
mRNA	✓	✓	✓	✓	✓
Small non-coding RNA	✓		✓		
Long polyA devoid RNA	✓		✓	✓	✓
Full-length cDNA	✓	✓			✓
UMIs	✓	✓			✓
Scalability/ Price	✓	✓		✓	✓

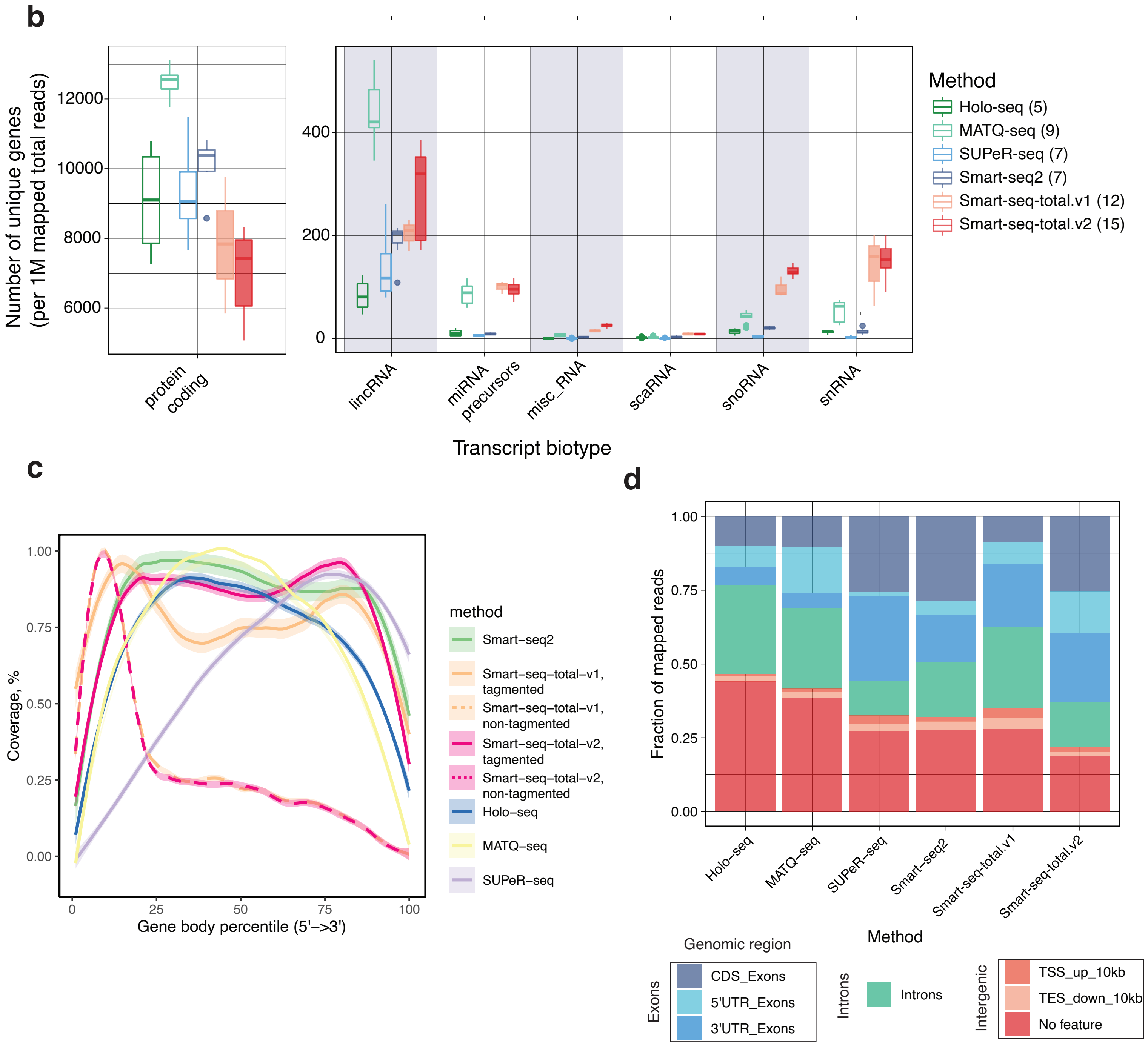

Supplementary figure 5

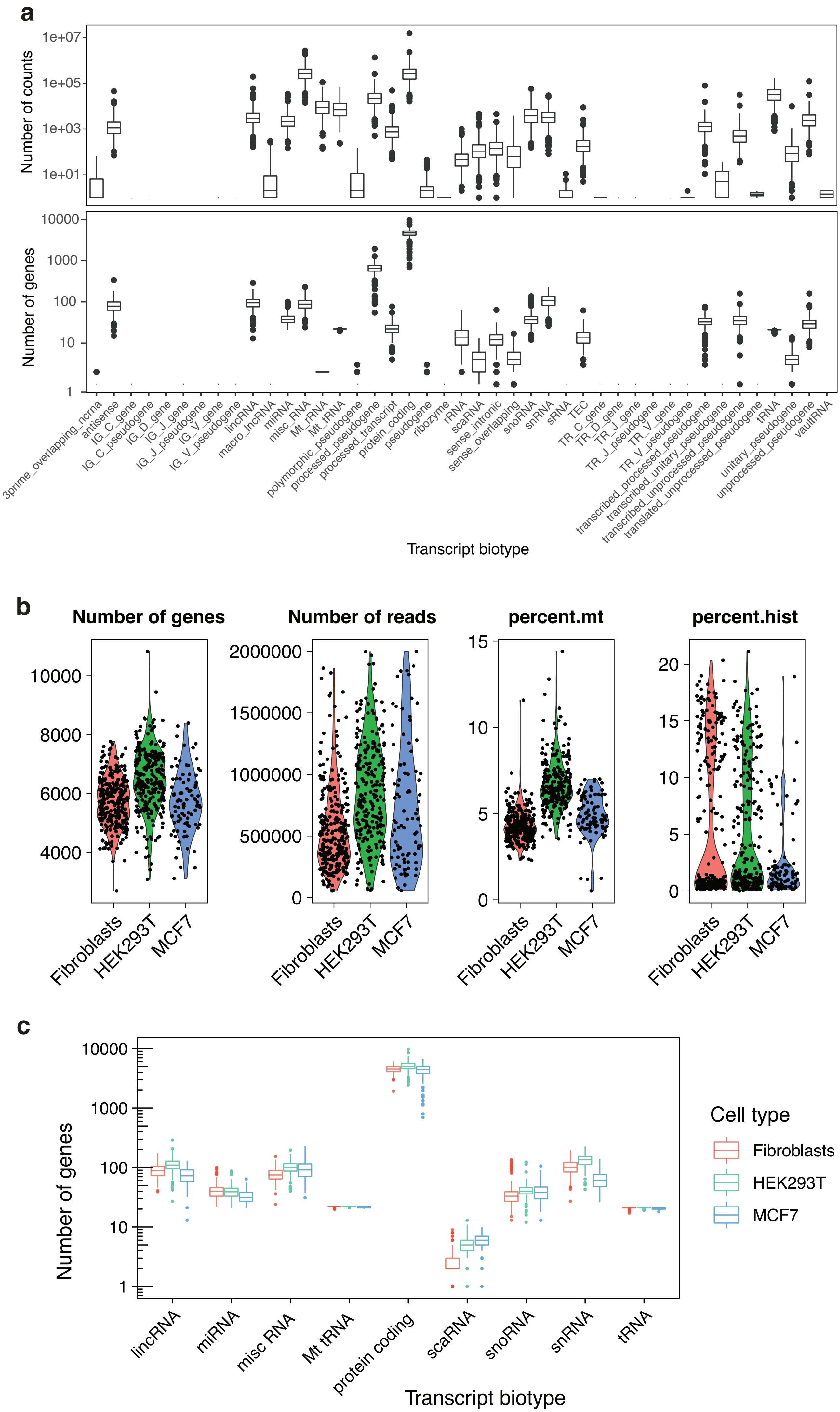

Supplementary figure 6

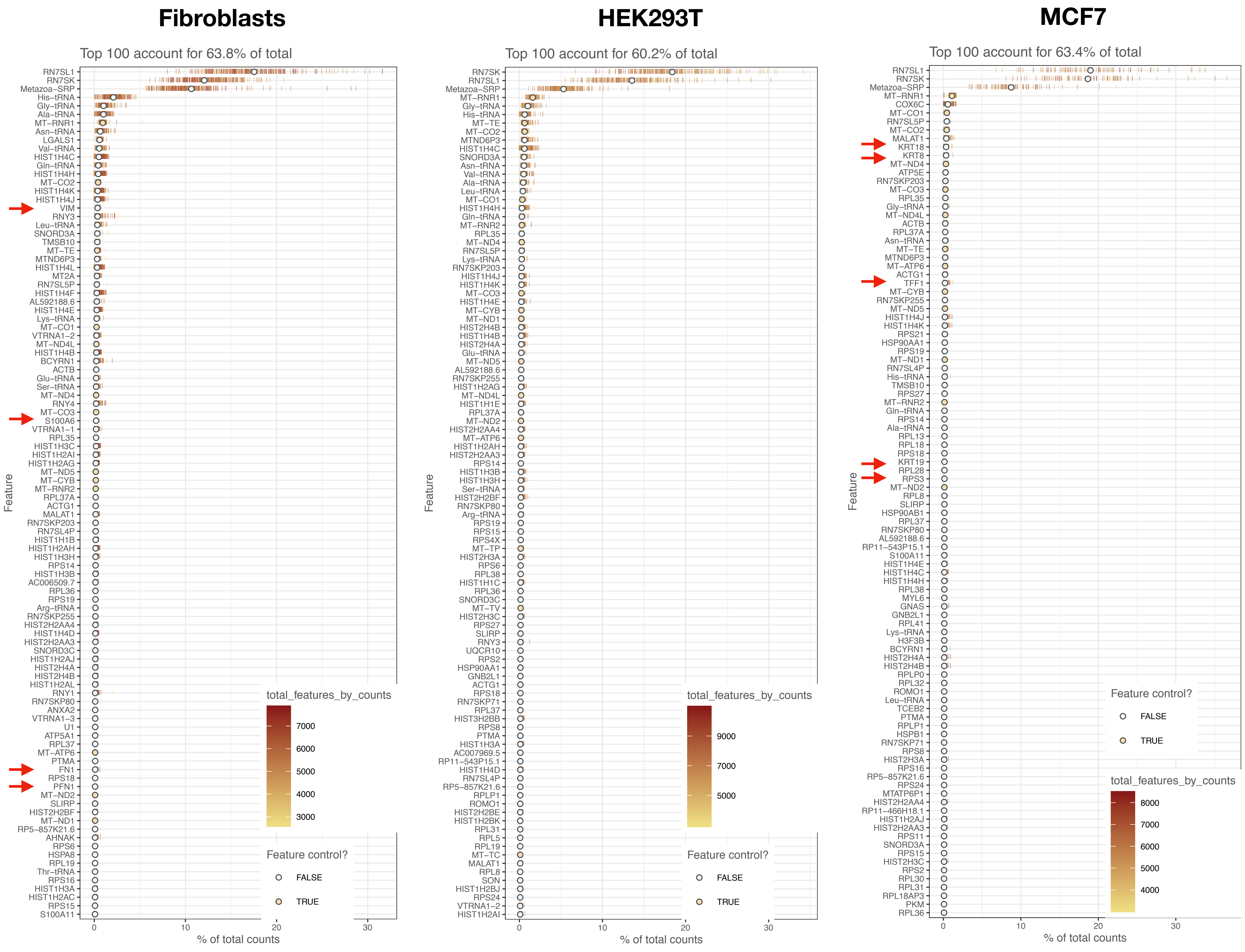

Supplementary figure 7

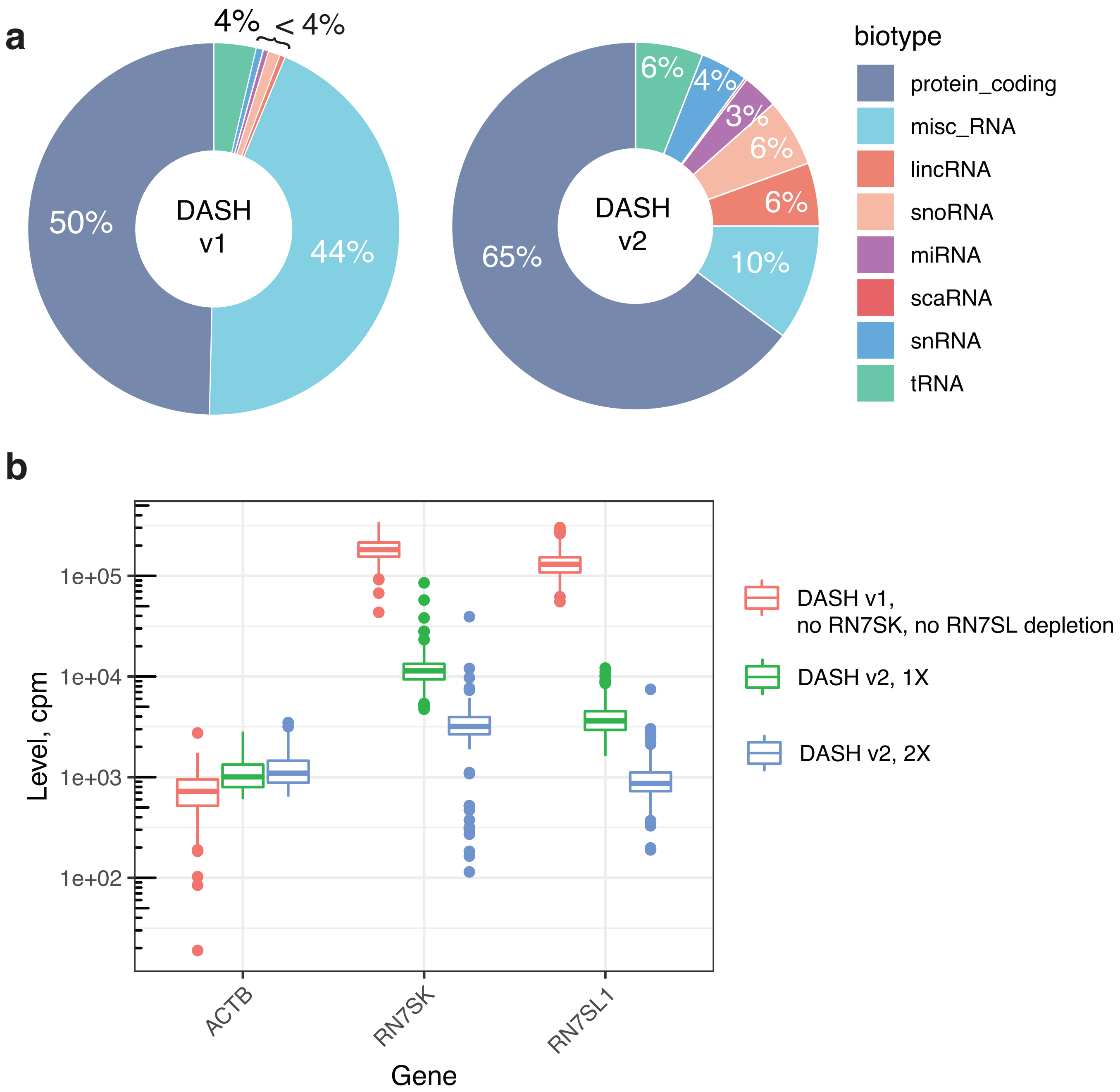

Supplementary figure 8

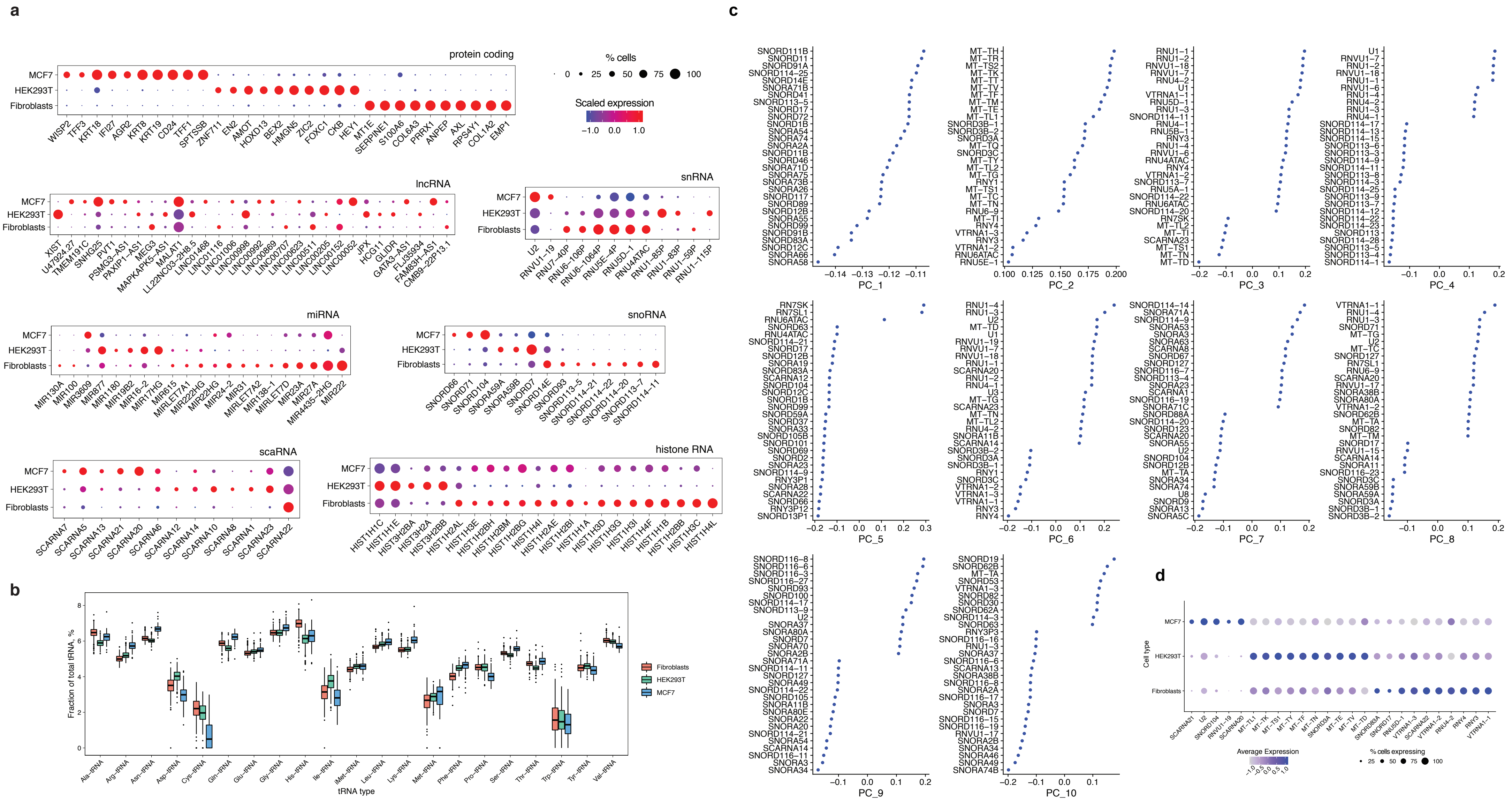

Supplementary figure 9

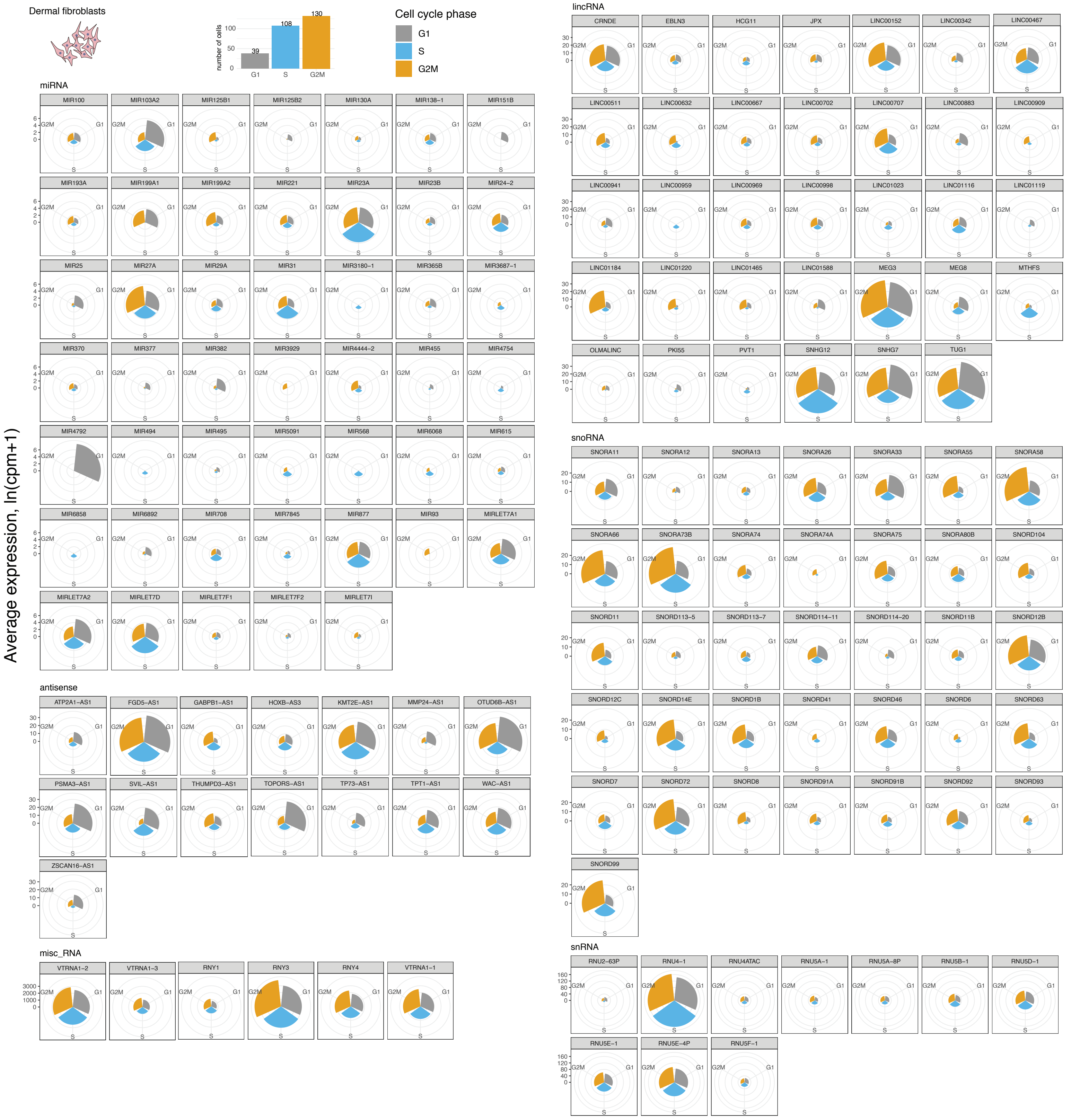

Supplementary figure 10

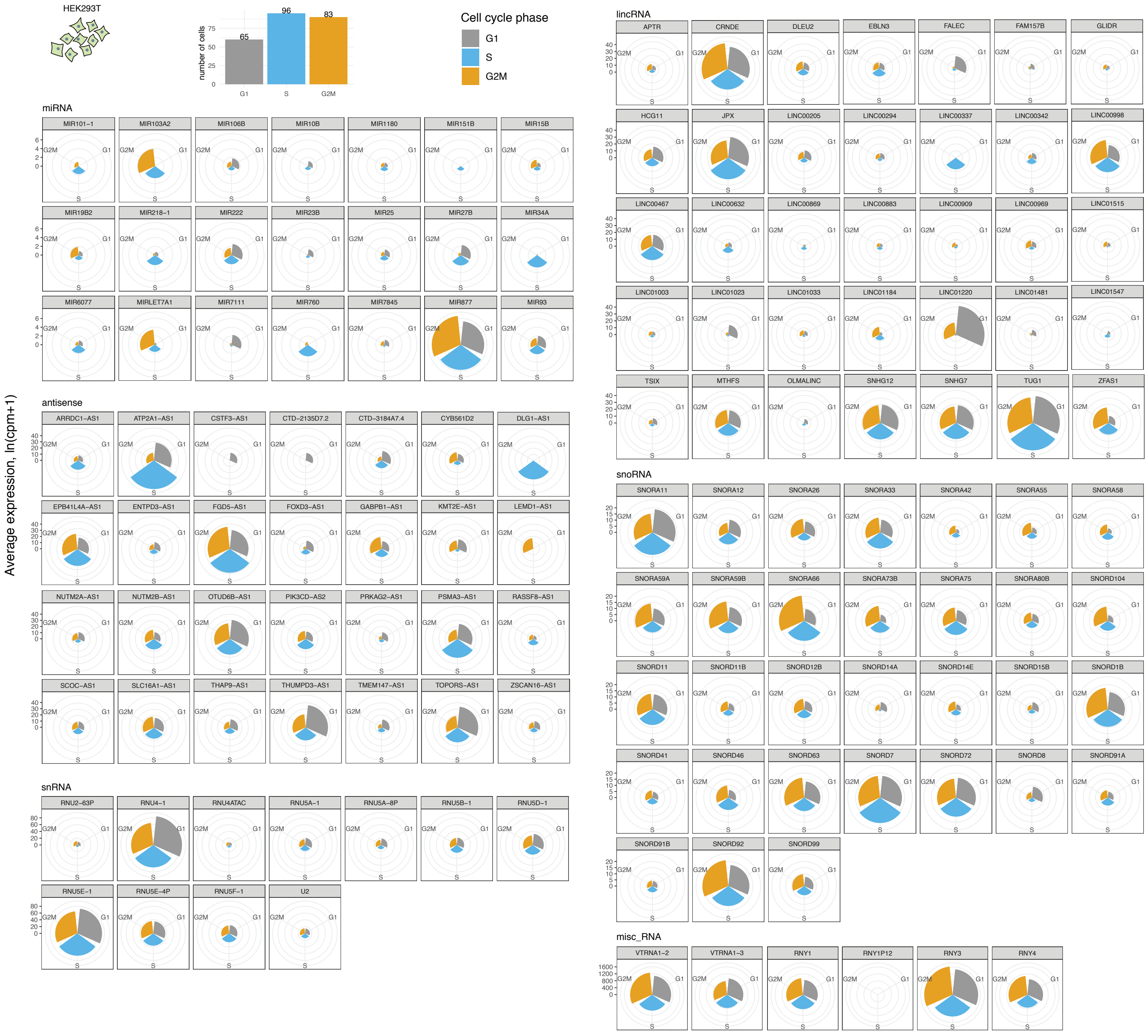

Supplementary figure 11

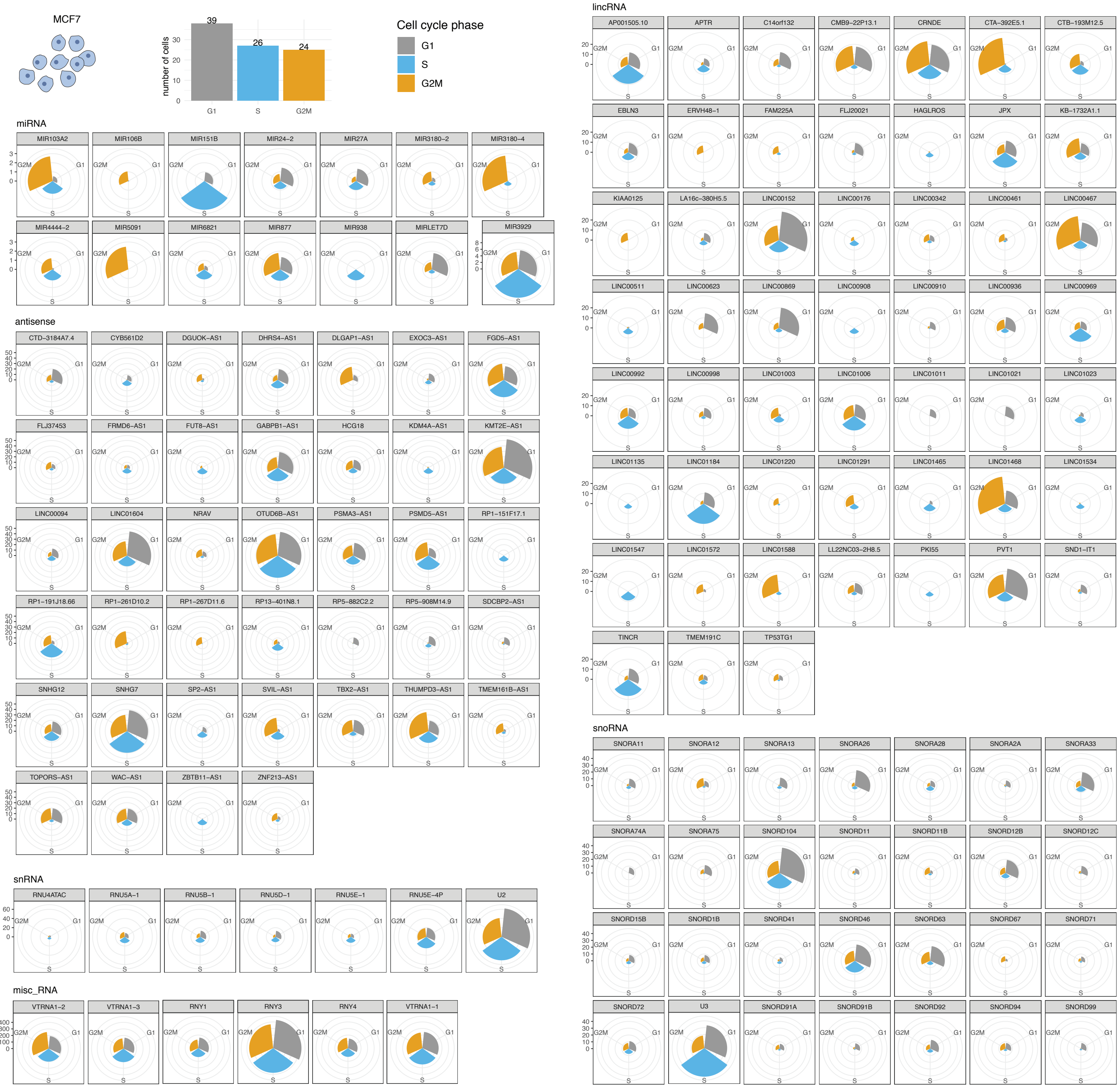

Supplementary figure 12

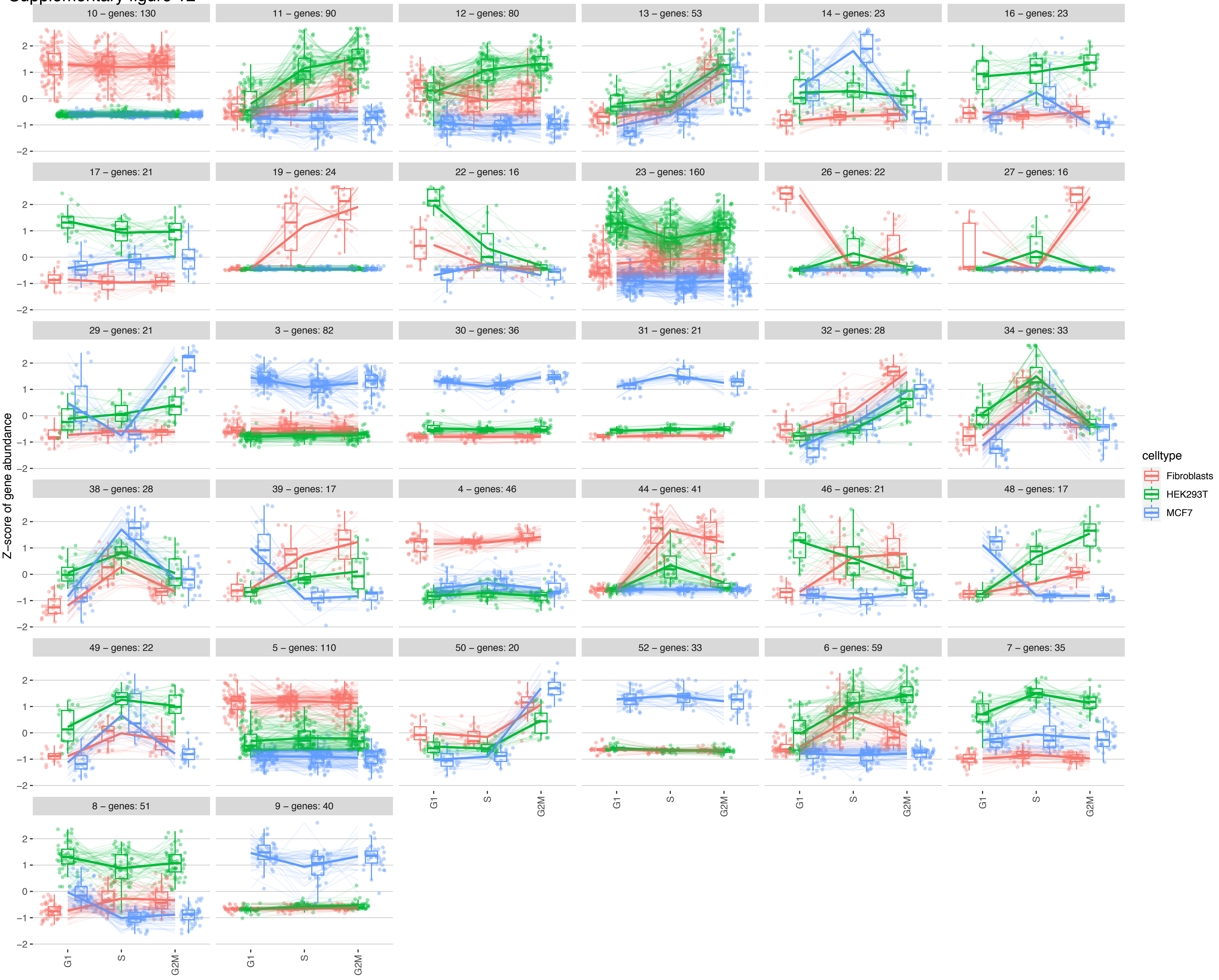

Supplementary figure 13

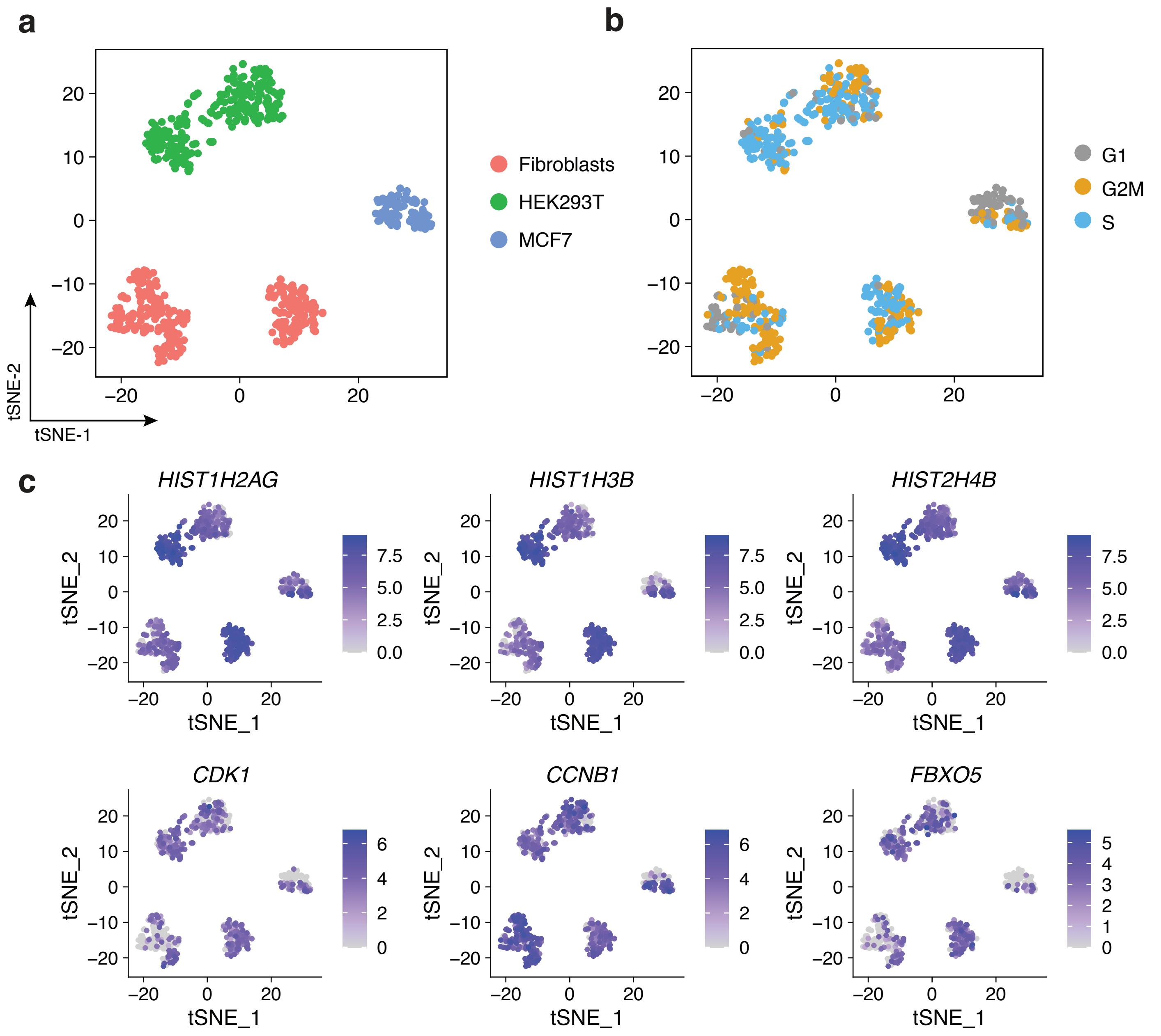

Supplementary figure 14

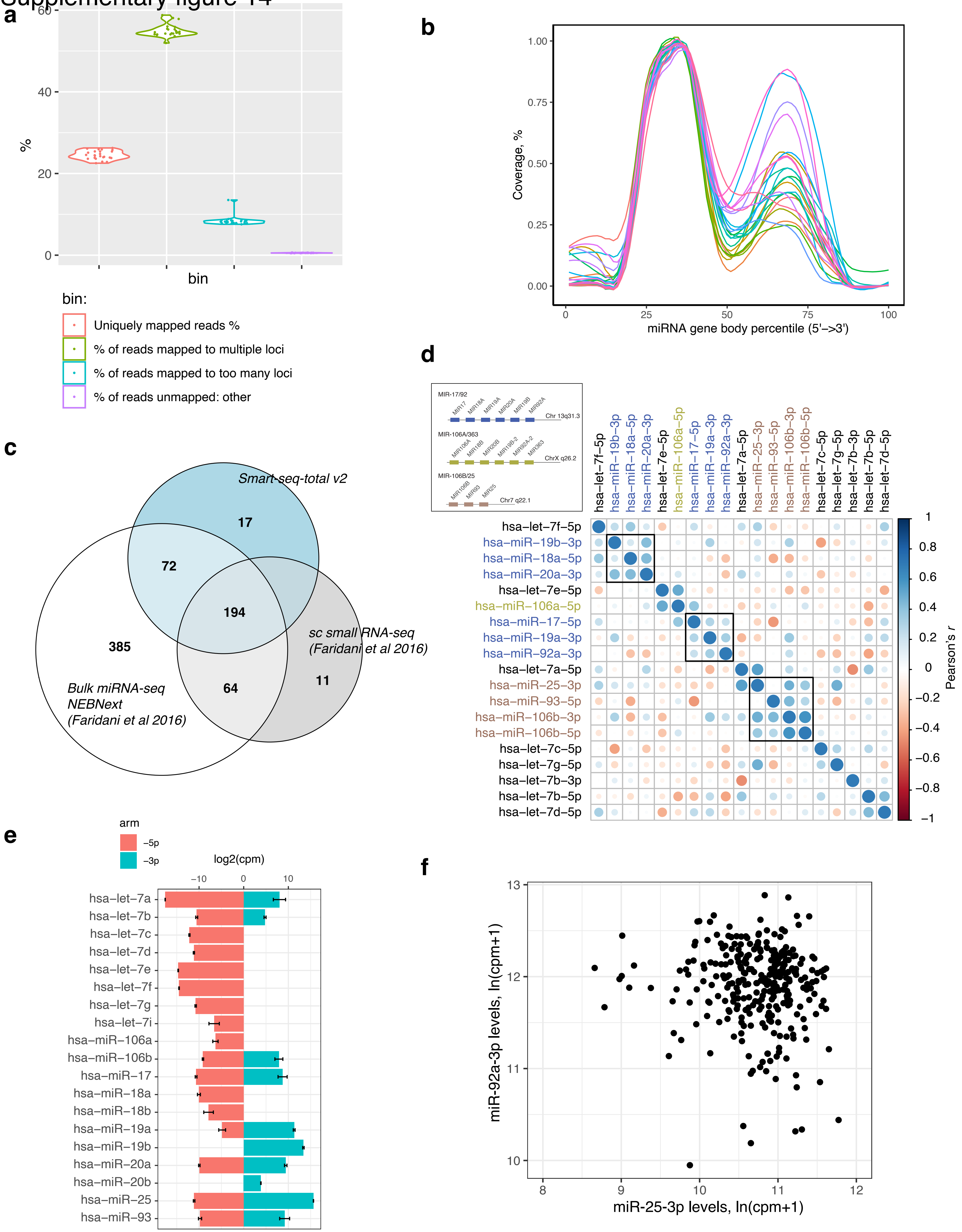

Supplementary figure 15

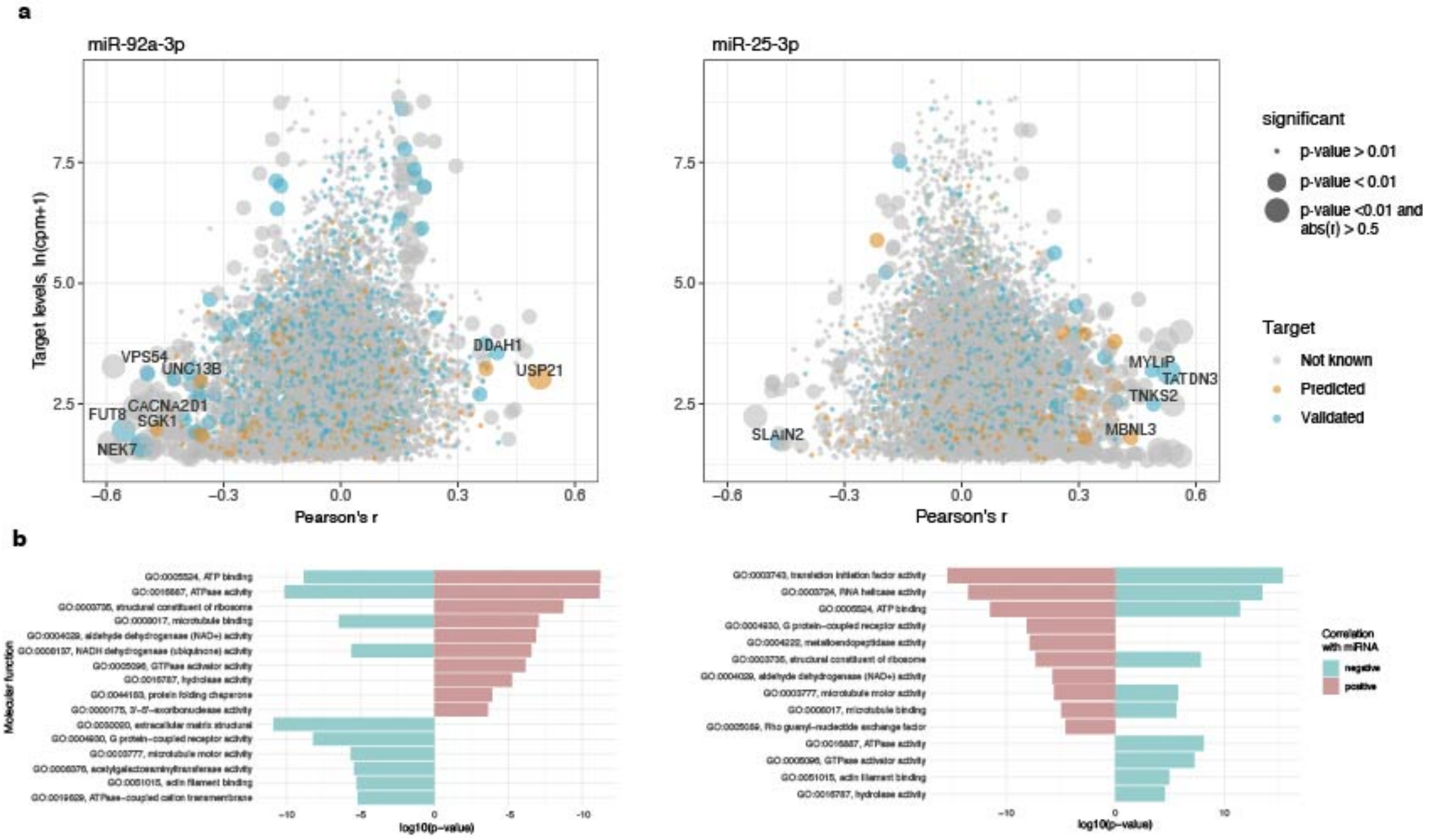

Supplementary figure 16

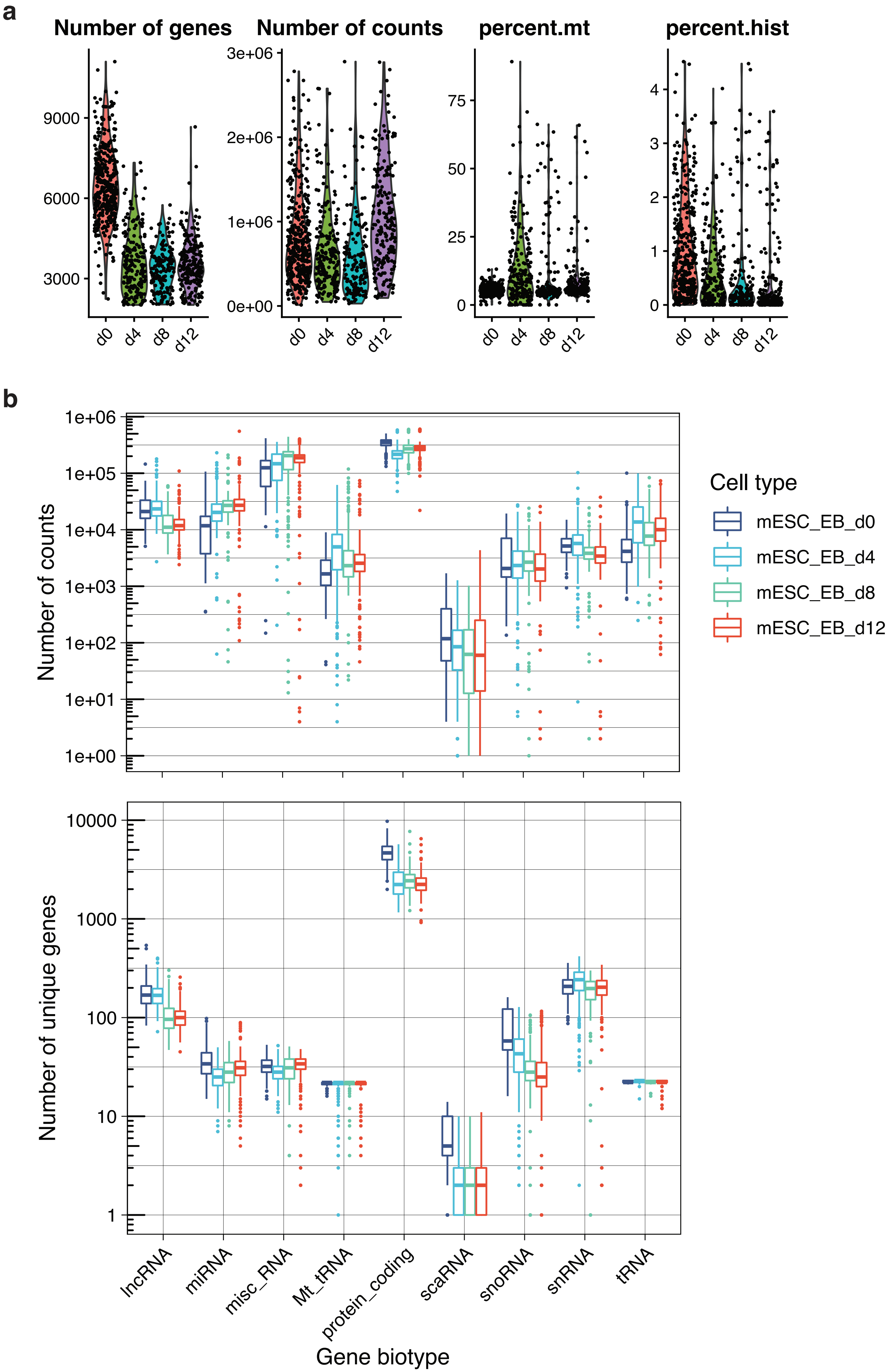

Supplementary figure 17

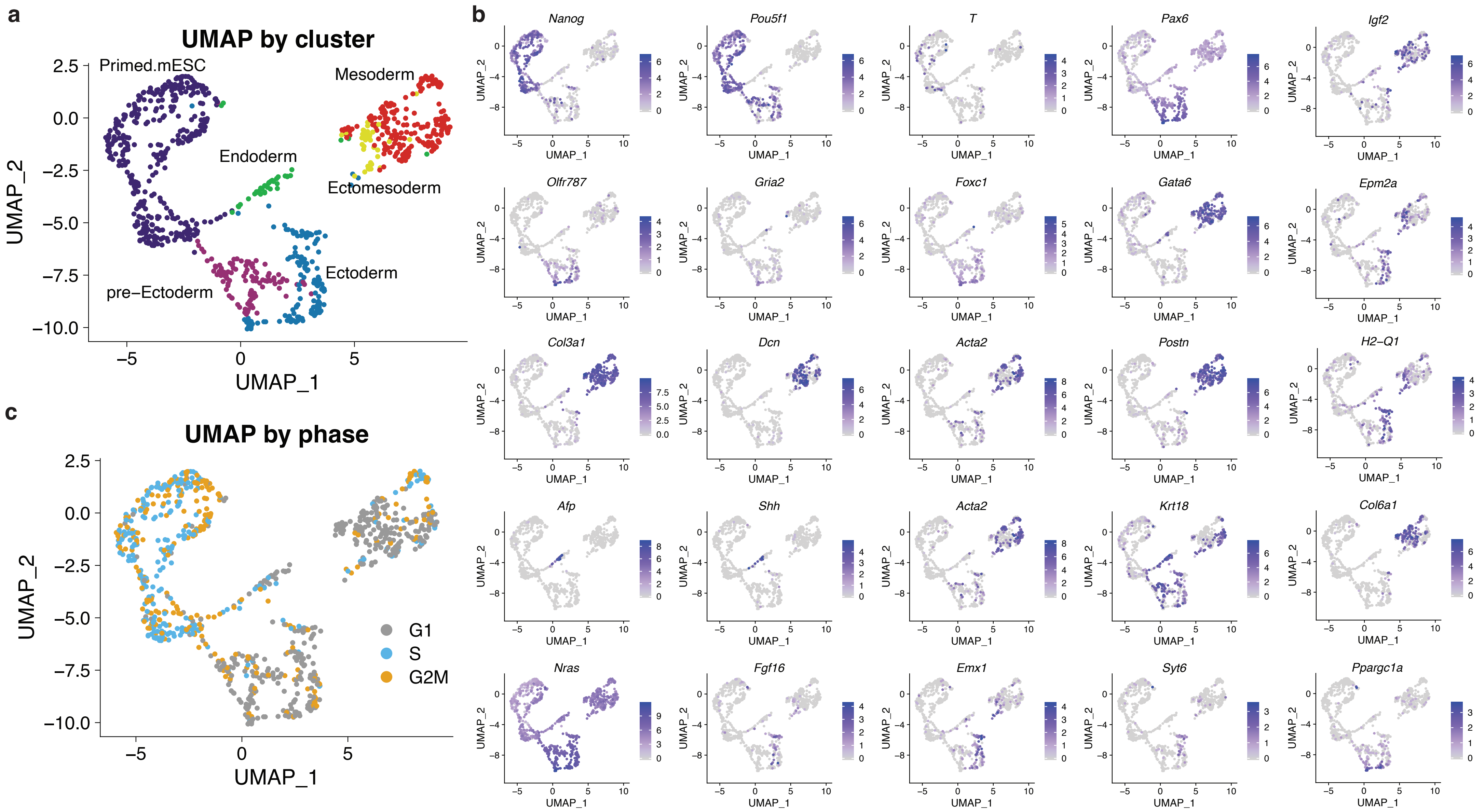

Supplementary figure 18

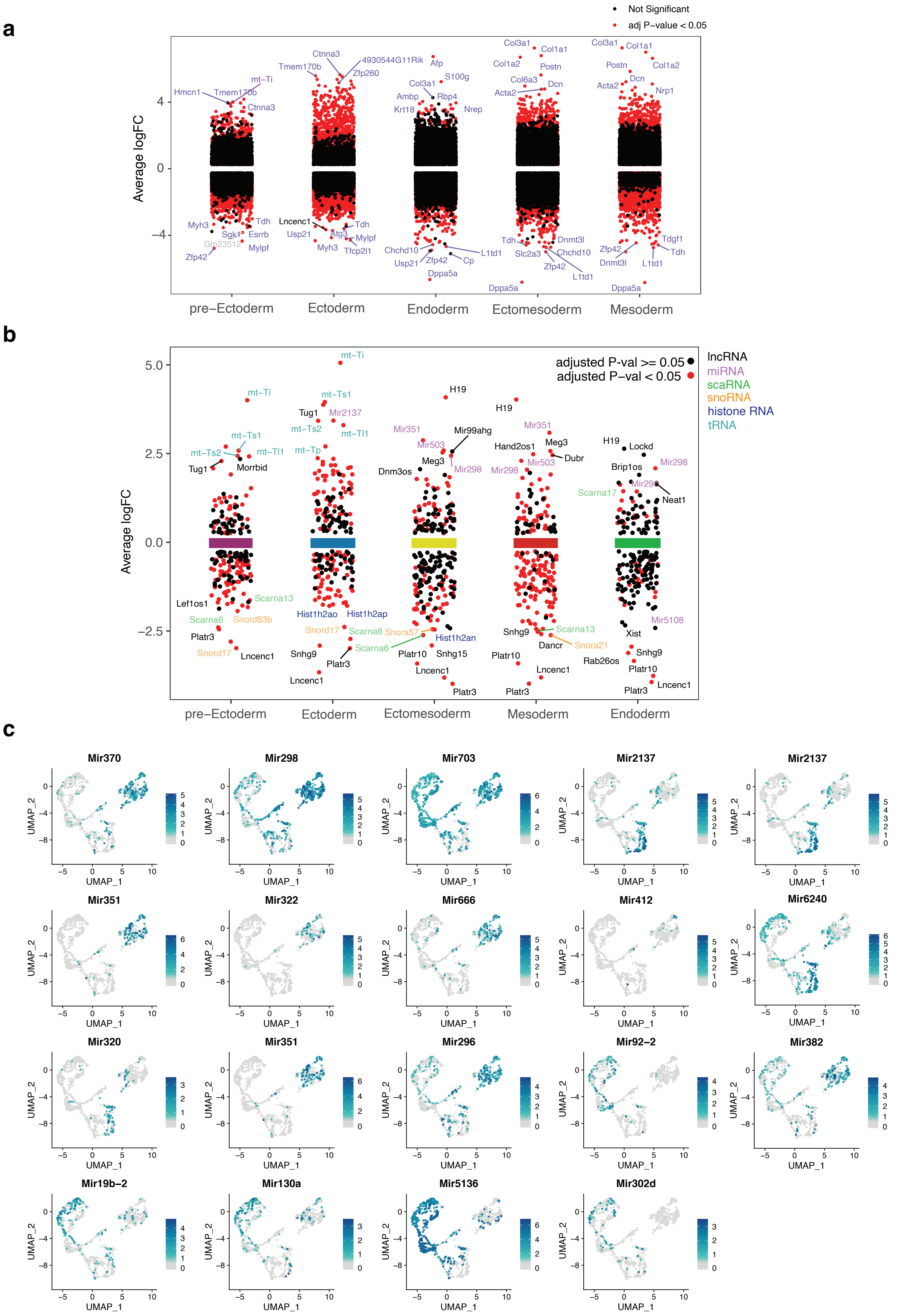

Supplementary figure 19

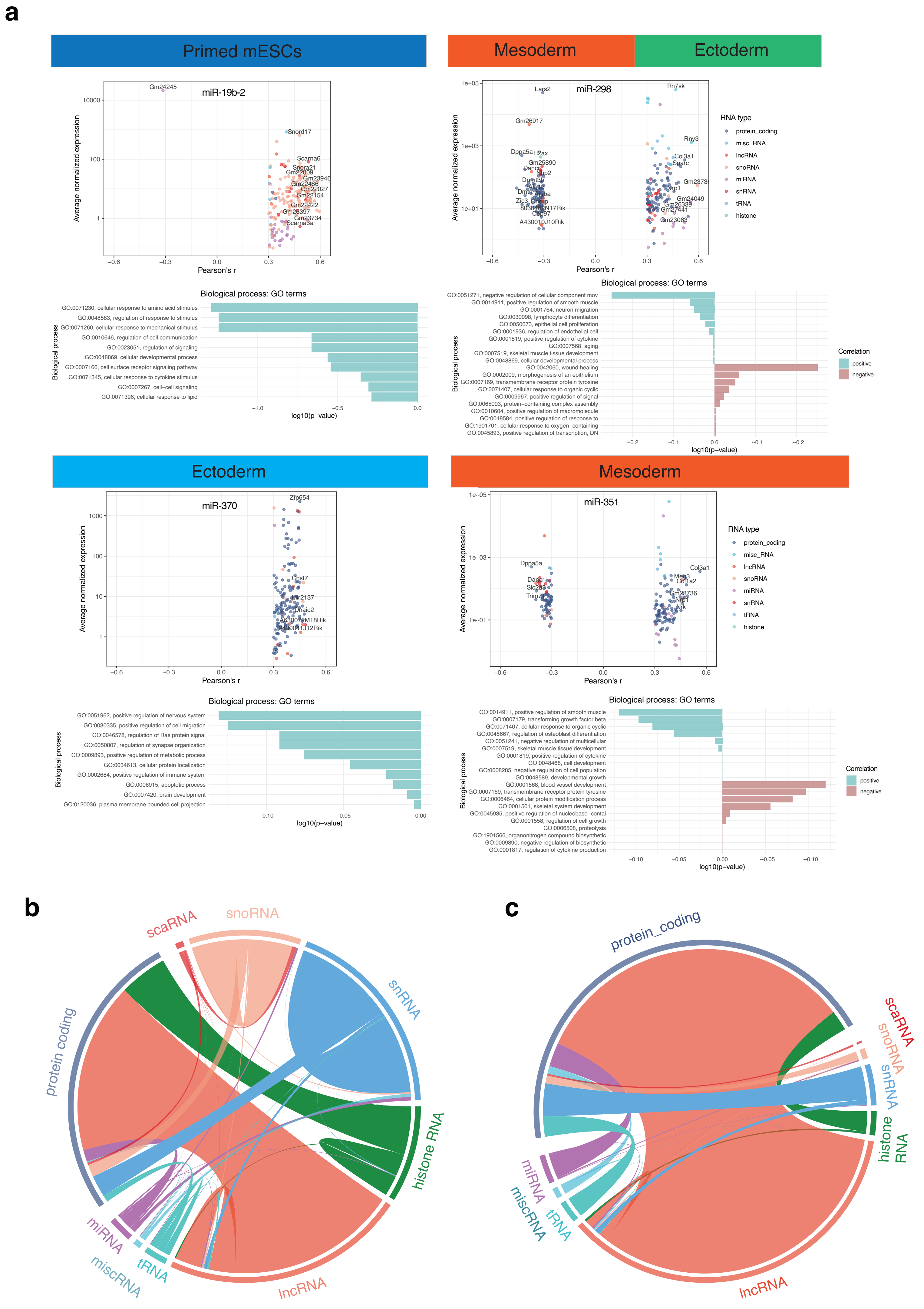

Supplementary table S1.

5X Cas9 Buffer Composition

Reagent	Stock Concentration	Final Concentration	50 mL Example
HEPES pH 7.4	1 M	100 mM	5 mL
KCl	2 M	500 mM	12.5 mL
$MgCl_2$	1 M	25 mM	1.25 mL
DTT	100 mM	5 mM	2.5 mL
Glycerol	100 %	25%	12.5 mL
Nuclease-Free H_20			16.25 mL
**Total**			**50 mL**

crRNA Guide Sequences v1:

Guide Name	Guide Sequences (5’-3’ excluding PAM sequence)
mm10_RN45s_crRNA1	UUUAACGAGGAUCCAUUGGA
mm10_RN45s_crRNA2	GUCUUGUGUGUGUCCUCGCC
mm10_RN45s_crRNA3	CCGCCUCGGGGGCGUGCGUU
mm10_RN45s_crRNA4	CGCGCCGGUUCGCGUCUGCU
mm10_RN45s_crRNA5	AGGCAUCGGUGUGUCGGCAU
mm10_RN45s_crRNA6	CGCCUGUGCGCCUGUGCGUA
mm10_RN45s_crRNA7	CGCCCGGUCGCGUUCGUGCC
mm10_RN45s_crRNA8	CAAAUUACCCACUCCCGACC
mm10_RN45s_crRNA9	GCCGUGUGCCCGCGCGGUGU
mm10_RN45s_crRNA10	GCUGGCCCGCGUCGCGGGUG

mm10_RN45s_crRNA11	GGUCUGGGGGCGUGCCCUCG
mm10_RN45s_crRNA12	CGGGUCUGUGUGCGCGUGUG
mm10_RN45s_crRNA13	CAUGCAUGUCUAAGUACGCA
mm10_RN45s_crRNA14	UGCCCUCACGUGUUUCACUU
mm10_RN45s_crRNA15	GGGUUUUUGACCCGUCCCGG
mm10_RN45s_crRNA16	GCUGGGGCGGUUGUCGCGUG
mm10_RN45s_crRNA17	UUCGGGCCCCUCCCGGUUGG
mm10_RN45s_crRNA18	UCGUAGUUGGAUCUUGGGAG
mm10_RN45s_crRNA19	GAGUUCGGGGAGGGAUCACG
mm10_RN45s_crRNA20	CCCUUCUCGCGCCUUCCCGU
mm10_RN45s_crRNA21	GAGGAAUUCCCAGUAAGUGC
mm10_RN45s_crRNA22	CUGACCUCGCCACCCUACCG
mm10_RN45s_crRNA23	UCCUUCCAUCUCUCGCGCAA
mm10_RN45s_crRNA24	CGUUAUUCCCAUGACCCGCC
mm10_RN45s_crRNA25	CGGACAUUCAUGGCGAAUGG
mm10_RN45s_crRNA26	GGGGUCGGUCUGGGUCCGUC
mm10_RN45s_crRNA27	CGGGGACGGGACCGUUCUGU

mm10_RN45s_crRNA28	UCCGAUAACGAACGAGACUC
mm10_RN45s_crRNA29	CUCUCCUACUUGGAUAACUG
mm10_RN45s_crRNA30	AAGGCAGGGGUGCGGCUCUC
mm10_RN45s_crRNA31	GUGGCGCUUCGUCGGGGUUC
mm10_RN45s_crRNA32	GCGGUUCGGGUGUGUGCUUG
mm10_RN45s_crRNA33	UUAUCAGAUCAAAACCAACC
mm10_RN45s_crRNA34	UUCGUCGUCCGCUCCGGGCG
mm10_RN45s_crRNA35	CUUUCUCUCUGUGGGCGGGU
mm10_RN45s_crRNA36	CUCUUUCUCGAUUCCGUGGG
mm10_RN45s_crRNA37	AGGGAAGUCGGUCGUUCGGG
mm10_RN45s_crRNA38	UCUGAGAAGCCCGUGAGAGG
mm10_RN45s_crRNA39	CAUUAAUCAAGAACGAAAGU
mm10_RN45s_crRNA40	CGGCUAGACGCGGGUGUCGC
mm10_RN45s_crRNA41	CGCGGCAGCGUUCCCACGGC
mm10_RN45s_crRNA42	CUGUGGUUUGGAGGGCGUCC
mm10_RN45s_crRNA43	UUUAGUGAGGCCCACGGCCC
mm10_RN45s_crRNA44	CGUGUCCCGGUGUGGCGGUG

mm10_RN45s_crRNA45	AGGUGUCGGAGAGCUGUCCC
mm10_RN45s_crRNA46	AGGUGUGGUGGGACUGCUCA
mm10_RN45s_crRNA47	ACCCGAGAUUGAGCAAUAAC
mm10_RN45s_crRNA48	AAAGGGAAAGAGGCUAGCAG
mm10_RN45s_crRNA49	GCCGCUAGAGGUGAAAUUCU
mm10_RN45s_crRNA50	GGAAUAAUGGAAUAGGACCG
mm10_RN45s_crRNA51	UUCGCUCGCGCUCCCUUACC
mm10_RN45s_crRNA52	UUUGAGAGGCCUGGCUUUCG
mm10_RN45s_crRNA53	CGUUGCAUACCCUUCCCGUC
mm10_RN45s_crRNA54	UGAGCCCCUGCCGCACCCGC
mm10_RN45s_crRNA55	ACCACGGGUGACGGGGAAUC
mm10_RN45s_crRNA56	GCUCUUAGCUGAGUGUCCCG
mm10_RN45s_crRNA57	CUAGGUGCCUGCUUCUGAGC
mm10_RN45s_crRNA58	UUCGGGUUUCCCGCGCCUGC
mm10_RN45s_crRNA59	UCCUCCCCGCUCGCCGCAGC
mm10_RN45s_crRNA60	UGGGGAGUGAAUGGUGCUAC
mm10_RN45s_crRNA61	UCUCGCGGGCGUCCGCGCGG

mm10_RN45s_crRNA62	UGUGGUGGUGGCUGGGGAGA
mm10_RN45s_crRNA63	CGGAAGGGCACCACCAGGAG
mm10_RN45s_crRNA64	GGUAGGCAACGGUGGGCUCC
mm10_RN45s_crRNA65	CCCGUUUUUGUUCCUUCCGC
mm10_RN45s_crRNA66	GCCGGGGGCUGGCCGCUGUC
mm10_RN5.8s_crRNA67	CGACACUUCGAACGCACUUG
mm10_RN5.8s_crNA68	ACUCUUAGCGGUGGAUCACU
mm10_RN5.8s_crRNA69	CGAGAAUUAAUGUGAAUUGC

crRNA Guide Sequences v2 (sequences used for the IDT order form):

**Cytoplasmic rRNA and miscRNA**	**Target Sequence**	Location in a plate
RNA7SK_1	GTCCATTTGTAGGAGAACGT	A01
RNA7SK_2	TCCAAATGAGGCGCTGCATG	A02
RNA7SK_3	ACCCTACGTTCTCCTACAAA	A03
RNA7SK2_1	CACTAAGTTCGGCATCAATA	A04
RNA7SK2_2	GACCACCAGGTTGCCTAAGG	A05
RNA7SK2_3	*GGAGTGCAGTGGCTATTCAC*	(optional)
7SL_1	CACTAAGTTCGGCATCAATA	A06
7SL_2	GACCACCAGGTTGCCTAAGG	A07
5.8S_1	CGACACTTCGAACGCACTTG	A08
5.8S_2	ACTCTTAGCGGTGGATCACT	A09
5S.1	TCTGATCTCGGAAGCTAAGC	A10
5S.2	CTCCCATCCAAGTACTAACC	A11
**mtRNA**		
MT-RNR1_1	TTGACCTAACGTCTTTACGT	B01

MT-RNR1_2	CACGAAATTGACCAACCCTG	B02
MT-RNR1_3	AGGGTGAACTCACTGGAACG	B03
MT-RNR1_4	AGTAGAGTGCTTAGTTGAAC	B04
MT-RNR1_5	GTAGTCTATTTTGTGTCAAC	B05
MT-RNR1_6	AGAGGAGACAAGTCGTAACA	B06
MT-RNR1_7	GGGTTTATCGATTACAGAAC	B07
MT-RNR2_1	CTGAACTCCTCACACCCAAT	B08
MT-RNR2_2	AAGGTTGTCTGGTAGTAAGG	B09
MT-RNR2_3	CCTCACTGTCAACCCAACAC	B10
MT-RNR2_4	GCCGTTAAACATGTGTCACT	B11
MT-RNR2_5	GCTGCTTTTAGGCCTACTAT	B12
MT-RNR2_6	TTGGACAACCAGCTATCACC	C01
MT-RNR2_7	CTGTTCTTAGGTAGCTCGTC	C02
MT-RNR2_8	TGAGATGATATCATTTACGG	C03
MT-RNR2_9	TGTTCCGTTGGTCAAGTTAT	C04
MT-RNR2_10	TTTGGTAGTTTAGGACCTGT	C05
MT-RNR2_11	CTGTATGAATGGCTCCACGA	C06
MT-RNR2_12	TTTAATAGCGGCTGCACCAT	C07
**18S and 28S**		
18S_1	TAGAGTCACCAAAGCCGCCG	D01
18S_2	CTCCAATGGATCCTCGTTAA	D02
18S_3	TAGAGCTAATACATGCCGAC	D03
18S_4	ACGGCTACCACATCCAAGGA	D04
18S_5	AATTACCCACTCCCGACCCG	D05
18S_6	CATGTCTAAGTACGCACGGC	D06
18S_7	CATGGTGACCACGGGTGACG	D07
18S_8	ACTCAGCTAAGAGCATCGAG	D08
18S_9	AGTCGTAACAAGGTTTCCGT	D09
18S_10	CATCCAATCGGTAGTAGCGA	D10
18S_11	CATCACGAATGGGGTTCAAC	D11
18S_12	GTTGAGTCAAATTAAGCCGC	D12
18S_13	ATTCTTGGACCGGCGCAAGA	E01
28S_1	CGGACAAACCCTTGTGTCGA	E02
28S_2	GGACGAAGGGCACTCCGCAC	E03
28S_3	CGGTTCCTCTCGTACTGAGC	E04
28S_4	CACTAATAGGGAACGTGAGC	E05
28S_5	ATTCGTAGACGACCTGCTTC	E06

28S_6	GGCTCCCAAACCACGCTCCC	E07
28S_7	GGTCGACCCGGGACACGTGC	E08
28S_8	CCGTCGGGACGAACCGCAAC	E09
28S_9	CTCGTGACCTCTCCTCGGTC	E10
28S_10	CCGTCTCGGTCGGCACTCCG	E11
28S_11	CTAAGTGTCAAGAGCCGAGA	E12
28S_12	GAGGAAGCATTACTGGCTGA	F01
28S_13	ACGGTGGAGCTGGGACCACG	F02

Supplementary table S2. Smart-seq-total primers used in the present study.

Smart-seq-total v1 oligo-dT	5’-Biotin-CATAGTCTCGTGGGCTCGGAGATGTGTATAAGAGACAGT30VN-3’
Smart-seq-total v1 TSO	5′-biotin-dUCGdUCGGCAGCGdUCAGdUdUGdUAdUCAACdUCAGACAdUrGrG+G-3′
Smart-seq-total v1 RV Amp	5'-GTCTCGTGGGCTCGGAGATGTG-3'
Smart-seq-total v1 FW Amp	5'-TCGTCGGCAGCGTCAGTTGTATCAACT-3'
Smart-seq-total v1 Custom SeqRead1	5’- TCGGCAGCGTCAGTTGTATCAACTCAGACATGGG-3’
Smart-seq-total v2 oligo-dT	5’-biotin-TCGTCGGCAGCGTCAGTTGTATCAACTCAGACNNNHNNNHNNNHNNNHNNGCTGTAACTTTTTTTTTTTTTTTTTTTTTTTVN
Smart-seq-total v2 TSO	5′-biotin-AdUGGCdUCGGAGAdUGdUGdUAdUAAGAGACAGdUCdUrGrG+G-3’
Smart-seq-total v2 RV cDNA Amp	5'-TCGTCGGCAGCGTCAGTTG-3’
Smart-seq-total v2 FW cDNA Amp	5'- GCTCGGAGATGTGTATAAGAGACAG-3’
Smart-seq-total v2 index Amp	5'- GTCTCGTGGGCTCGGAGATGTGTATAAGAGACAGTC-3’
Smart-seq-total v2 Custom Read1	5’-TCGGCAGCGTCAGTTGTATCAACTCAGACATGGG-3’
Smart-seq-total v2 Custom Index Read2	5’-GTCTGAGTTGATACAACTGACGCTGCCGACGA-3’

Supplementary table S3. Smart-seq-total cost estimate.

Step		Cost per cell	Cumulative cost per cell
1b	Lysis Plate	$ 0.10	$ 0.10
2	Sorted Plate of Cells	$ 0.02	$ 0.12
4a	polyA + RT Reaction	$ 0.21	$ 0.33
4b	DNA Preamp Plate	$ 0.11	$ 0.44
5b	Cleanup (BRAVO)	$ 0.38	$ 0.82
6	Quantified DNA Plate	$ 0.59	$ 1.41
7a	Normalized Lib Plate	$ 0.09	$ 1.50
8	Tagmentation + amplification	$ 0.05	$ 1.55
9	Pool, CRISPR-mediated depletion	$ 0.20	$ 1.75
10	Pool, Cleanup (Manual)	$ 0.02	$ 1.77
	Total:		$ 1.77

References

1. Gu, W. *et al.* Depletion of Abundant Sequences by Hybridization (DASH): using Cas9 to remove unwanted high-abundance species in sequencing libraries and molecular counting applications. *Genome Biol* **17**, 41 (2016).
2. Wang, L., Wang, S. & Li, W. RSeQC: quality control of RNA-seq experiments. *Bioinformatics* **28**, 2184–2185 (2012).
3. Svensson, V. *et al.* Power analysis of single-cell RNA-sequencing experiments. *Nat Methods* **14**, 381–387 (2017).
4. Bagnoli, J. W. *et al.* Sensitive and powerful single-cell RNA sequencing using mcSCRB-seq. *Nat Commun* **9**, 2937 (2018).
5. Faridani, O. R. *et al.* Single-cell sequencing of the small-RNA transcriptome. *Nat Biotechnol* **34**, 1264–1266 (2016).
